# Analysis of Gene Expression in 3D Spheroids Highlights a Survival Role for ASS1 in Mesothelioma

**DOI:** 10.1371/journal.pone.0150044

**Published:** 2016-03-16

**Authors:** Dario Barbone, Loes Van Dam, Carlo Follo, Puthen V. Jithesh, Shu-Dong Zhang, William G. Richards, Raphael Bueno, Dean A. Fennell, V. Courtney Broaddus

**Affiliations:** 1 Department of Medicine, Pulmonary, San Francisco General Hospital, University of California San Francisco, San Francisco, California, United States of America; 2 Department of Molecular Cancer Research, Center for Molecular Medicine, University Medical Center Utrecht, Utrecht, The Netherlands; 3 Sidra Medical and Research Center, Doha, Qatar; 4 Center for Cancer Research and Cell Biology, Queen's University of Belfast, Belfast, United Kingdom; 5 Division of Thoracic Surgery, Brigham and Women's Hospital, Boston, Massachusetts, United States of America; 6 Department of Cancer Studies, Cancer Research UK Leicester Centre, University of Leicester, Leicester, United Kingdom; National Cancer Institute, NIH, UNITED STATES

## Abstract

To investigate the underlying causes of chemoresistance in malignant pleural mesothelioma, we have studied mesothelioma cell lines as 3D spheroids, which acquire increased chemoresistance compared to 2D monolayers. We asked whether the gene expression of 3D spheroids would reveal mechanisms of resistance. To address this, we measured gene expression of three mesothelioma cell lines, M28, REN and VAMT, grown as 2D monolayers and 3D spheroids. A total of 209 genes were differentially expressed in common by the three cell lines in 3D (138 upregulated and 71 downregulated), although a clear resistance pathway was not apparent. We then compared the list of 3D genes with two publicly available datasets of gene expression of 56 pleural mesotheliomas compared to normal tissues. Interestingly, only three genes were increased in both 3D spheroids and human tumors: argininosuccinate synthase 1 (ASS1), annexin A4 (ANXA4) and major vault protein (MVP); of these, ASS1 was the only consistently upregulated of the three genes by qRT-PCR. To measure ASS1 protein expression, we stained 2 sets of tissue microarrays (TMA): one with 88 pleural mesothelioma samples and the other with additional 88 pleural mesotheliomas paired with matched normal tissues. Of the 176 tumors represented on the two TMAs, ASS1 was expressed in 87 (50%; staining greater than 1 up to 3+). For the paired samples, ASS1 expression in mesothelioma was significantly greater than in the normal tissues. Reduction of ASS1 expression by siRNA significantly sensitized mesothelioma spheroids to the pro-apoptotic effects of bortezomib and of cisplatin plus pemetrexed. Although mesothelioma is considered by many to be an ASS1-deficient tumor, our results show that ASS1 is elevated at the mRNA and protein levels in mesothelioma 3D spheroids and in human pleural mesotheliomas. We also have uncovered a survival role for ASS1, which may be amenable to targeting to undermine mesothelioma multicellular resistance.

## Introduction

Uncovering the causes of chemoresistance of tumors may help to develop effective therapies aimed at undermining their survival strategies. Solid tumors are characterized by a 3D environment that may help cancer cells acquire new biological properties, such as resistance to cell death and independence from growth factors and nutrients. Indeed, malignant mesothelioma, a 3D mass that develops from a 2D pleural monolayer, may derive some of its chemoresistance from its 3D morphology [1, 2]. Interestingly, the presence of 3D aggregates (spheroids) in pleural fluid is considered a characteristic of mesothelioma [3]. We believe that to devise improved treatments for malignant mesothelioma, it may be helpful to understand how the 3D environment supports its chemoresistance.

We have previously shown that mesothelioma cells acquire additional resistance to apoptosis when grown in 3D, a property termed multicellular resistance. To date we have found that multicellular resistance can be overcome by interfering with the mTOR pathway and the Bcl-2 repertoire, either by inhibition of anti-apoptotic proteins [4] or by activation of pro-apoptotic ones [5]. Here, we asked whether a specific genetic signature, expressed by cells in 3D, could offer insights into the molecular reprogramming that mediates additional apoptotic resistance. It is known that spheroids alter their gene expression pattern when compared to their monolayer counterparts [6]; nonetheless, a connection to multicellular resistance has not yet emerged.

Several studies have described the gene expression profile of mesothelioma [7–10]. It is well known that mesothelioma is characterized by NF2 and BAP1 loss [11] and that there is prognostic and diagnostic value in specific gene patterns [12–15]; nonetheless, no signature correlated to a specific chemoresistance pathway has been found. To answer this question, we have utilized 3D models to determine which genes are potentially implicated in multicellular resistance. To narrow down our results and identify genes that are clinically relevant, we compared our 2D-3D dataset with gene expression profiles of patient tumors compared to normal tissues.

In the present work, we demonstrate for the first time that ASS1 is upregulated in mesothelioma 3D spheroids, is expressed in mesothelioma tumor samples, and exhibits a survival role. Initially, this result was surprising, because mesothelioma is described in the literature as an ASS1-deficient tumor [16–20], whose auxotrophy for arginine can be targeted therapeutically [18]. Nonetheless, our data shows that ASS1 is a mesothelioma gene, as also described by Melaiu and colleagues [9, 10], is upregulated when mesothelioma cells, but not normal mesothelial cells, are grown as 3D spheroids, and that reduction of ASS1 protein levels has potential therapeutic value.

## Results

### Gene-expression changes found in 3D were associated with multiple pathways

**Fig 1:**
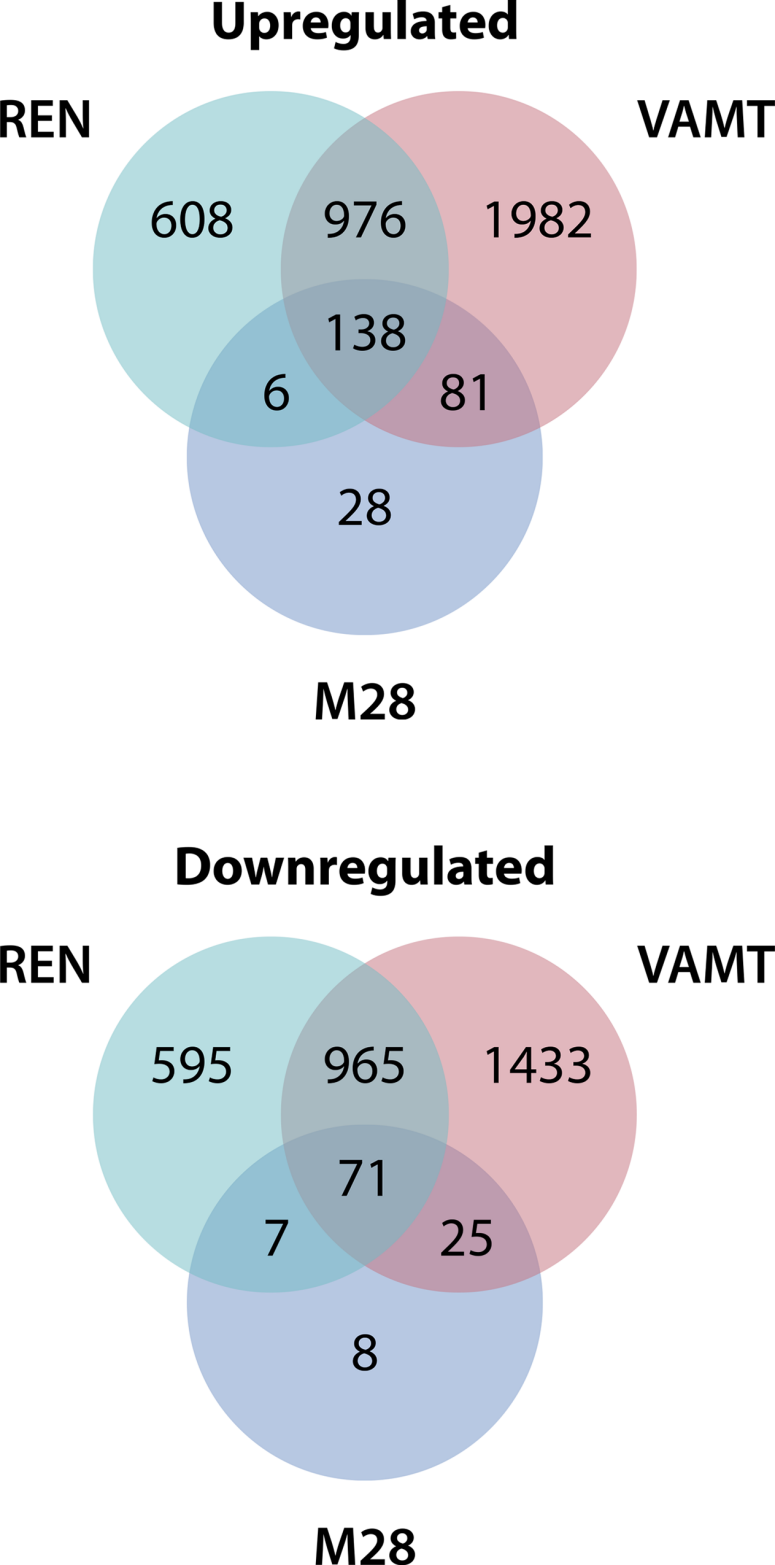
More than 200 genes are differentially expressed in mesothelioma spheroids compared to monolayers. Venn diagrams showing the number of genes differentially expressed by spheroids of three mesothelioma cell lines M28, REN and VAMT, compared to respective monolayers. The diagram also shows the number of genes upregulated (138) and downregulated (71) in common by the three cell lines.

To identify genes that are differentially expressed between monolayers and spheroids, we performed a microarray analysis of three mesothelioma cell lines (two epithelial, M28 and REN, and one sarcomatous, VAMT) grown in 2D and in 3D. We identified a total of 209 differentially expressed genes in all cell lines (Fig 1), of which 138 were upregulated (S1 Table) and 71 were downregulated (S2 Table). We used the Ingenuity Pathway Analysis platform to investigate the functional associations of these 209 differentially expressed genes. Among the top enriched molecular and cellular functions were pathways of cellular growth and differentiation, cellular development, and cell, tumor and tissue morphology (S3 Table). These results were confirmed by analysis using the Database for Annotation, Visualization and Integrated Discovery (DAVID), which showed the most significant enrichment in regulation of cell proliferation (GO:0042127) and wound healing (GO:0042060) (S4 Table). Although the pathways affected by the differentially expressed genes might be associated with chemoresistance, it was difficult to know how to study these pathways further. Instead, we decided to narrow the list of differentially expressed genes to identify those 3D mesothelioma genes with potential relevance in patients.

### ASS1, ANXA4 and MVP are elevated in both 3D spheroids and patients

To uncover clinically relevant genes, we cross-referenced our list of differentially expressed genes to two publicly available datasets (Gordon et al.,[21]—Singhal et al.,[22]) comprising the gene expression profiles of 56 mesotheliomas (40 and 16 respectively) compared to 14 normal tissue samples (9 and 5, respectively). By comparing these datasets, we found 3 genes that were elevated in 3D compared to 2D as well as in mesothelioma compared to normal tissue: argininosuccinate synthetase 1 (ASS1), annexin A4 (ANXA4) and the major vault protein (MVP).

**Fig 2:**
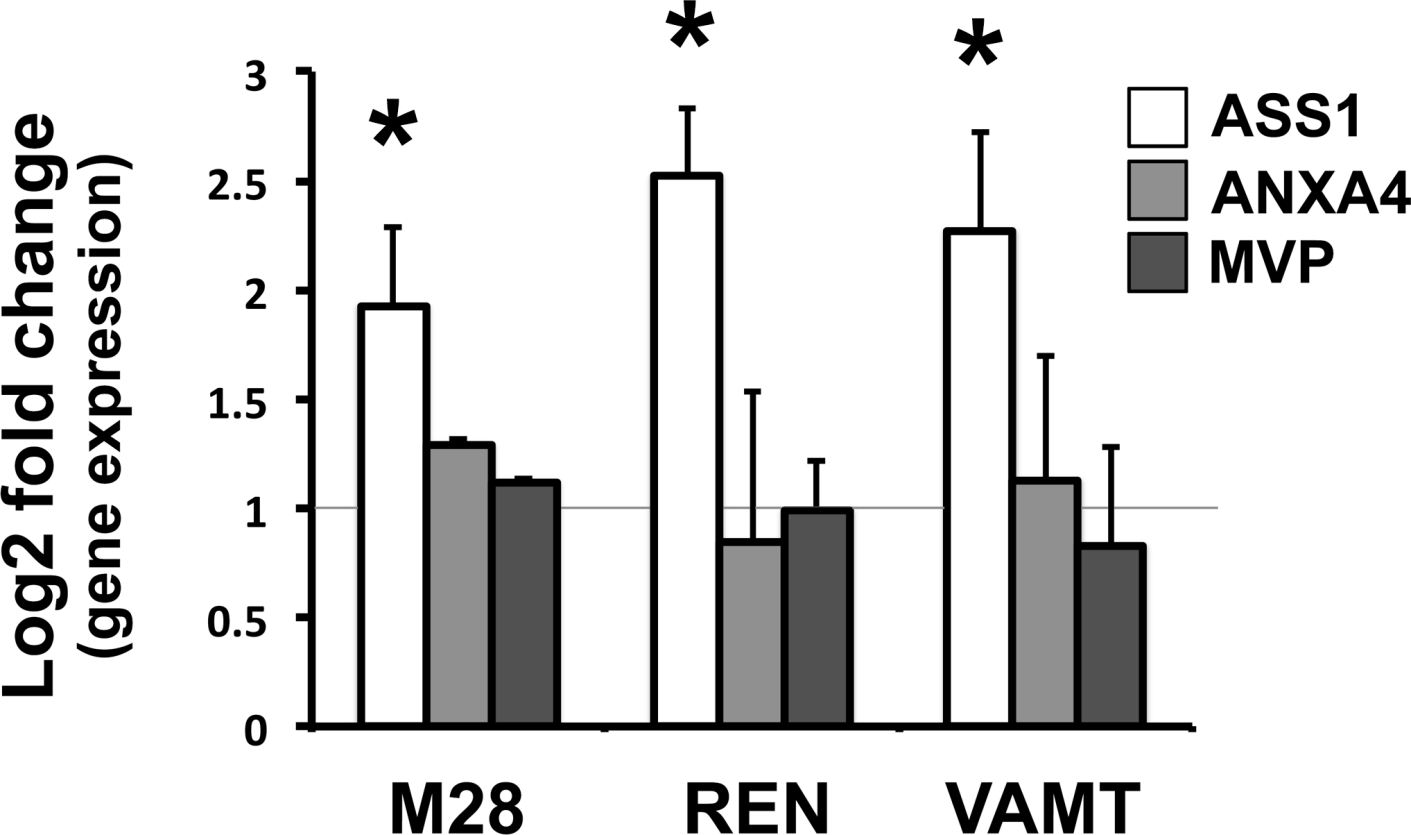
ASS1 gene expression is upregulated in 3D spheroids and mesothelioma patients. qRT-PCR results showing the Log2-transformed fold change in gene expression for ASS1, ANXA4 and MVP between M28, REN and VAMT spheroids and monolayers. Results were normalized to the gene expression levels of monolayers (*gray line*). Only ASS1 was upregulated in 3D in all three cell lines. (* *p* ≤ 0.05 n = 3).

We then validated the up-regulation of these genes by qRT-PCR. Of the three genes, ASS1 was the only gene confirmed to be upregulated in the three cell lines, M28, REN and VAMT (Fig 2). Interestingly, ASS1 is thought to be deficient in mesothelioma. In fact, many publications describe mesothelioma as an ASS1-deficient tumor [16–20], and thus one that can be targeted with arginine deiminase (ADI), an enzyme that degrades arginine [18]. Hence, to confirm our findings, we analyzed ASS1 expression at the protein level in both mesothelioma spheroids and patient samples.

### ASS1 protein is increased in 3D spheroids and expressed in tissues

**Fig 3:**
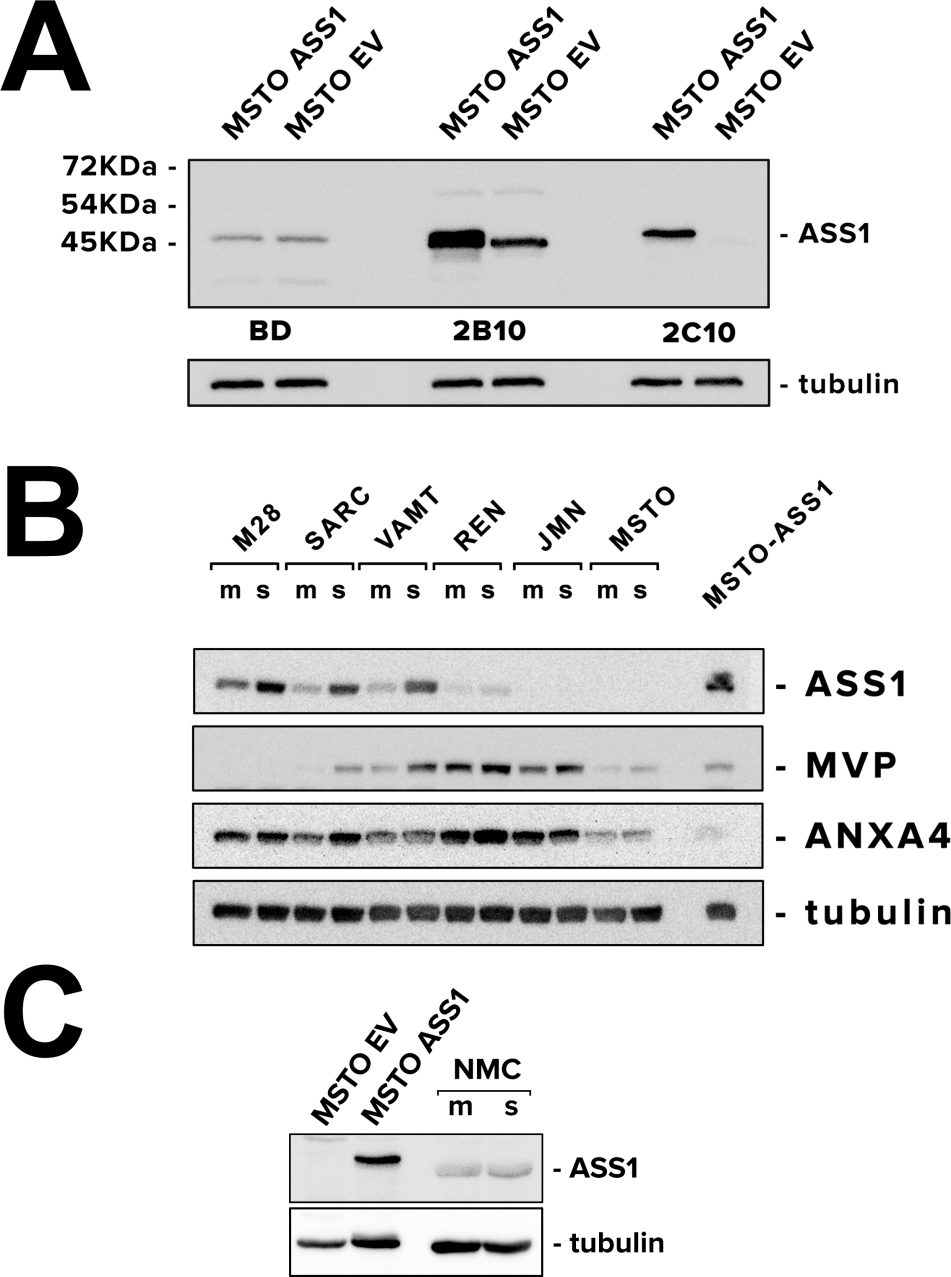
ASS1 protein is upregulated in 3D in mesothelioma cells but not in normal mesothelial cells. **(A)** ASS1 antibodies (BD clone 25, Sigma clone 2B10, and Millipore clone 2C10) were tested against ASS1-positive MSTO pIRES-ASS1 (as a positive control) and ASS1-negative MSTO pIRES-EV (as a negative control). Monoclonal antibody clone 2C10 provides the most accurate detection of ASS1 protein levels. **(B)** Immunoblot analysis of ASS1, ANXA4 and MVP in monolayers (m) and spheroids (s) grown from 6 mesothelioma cell lines (M28, SARC, VAMT, REN, JMN) and MSTO-211H (known to be ASS1-negative). MSTO pIRES-ASS1 cells were used as positive control for ASS1. ASS1 was the most consistently upregulated in 3D among the panel of cells tested. JMN and MSTO cells showed no ASS1 expression. **(C)** Immunoblot analysis of ASS1 in normal mesothelial cells grown as monolayers and spheroids. ASS1-negative MSTO pIRES-EV and ASS1-positive MSTO pIRES-ASS1 were used as controls for ASS1. Normal mesothelial cells did not upregulate ASS1 when grown in 3D.

To confirm the upregulation of ASS1 protein levels in spheroids, we investigated various antibodies because the antibody that has been widely used for the detection of ASS1 had been discontinued (BD, clone 25). Hence, we tested a panel of antibodies currently available for ASS1: the discontinued clone 25 [23] and newer clones 2B10 and 2C10. As positive and negative controls for ASS1, we used MSTO-211H transduced with ASS1 (MSTO-pIRES-ASS1) and with an empty vector (MSTO-pIRES-EV). We found that staining for both clone 25 and clone 2B10 was non-specific (especially BD clone 25), while clone 2C10 correctly identified ASS1 in transduced MSTO cells (Fig 3A). Therefore, we used clone 2C10 ASS1 antibody for detecting ASS1 protein in spheroids and in tumor.

We performed immunoblots on monolayers and spheroids generated from M28, REN and VAMT cells, the cell lines used in our microarray analysis. To these, we added three more cell lines (SARC, JMN and MSTO-211H), one of these known to be ASS1-negative (MSTO-211H). Four of the cell lines showed increased ASS1 protein levels in 3D compared to 2D, while two of them (JMN and MSTO-211H) lacked expression of ASS1 altogether (Fig 3B). Our findings suggest that cells capable of expressing ASS1 elevate its protein levels in 3D, whereas the cells with ASS1 deficiency do not. Interestingly, normal mesothelial cells did not upregulate ASS1 in 3D (Fig 3C).

**Fig 4:**
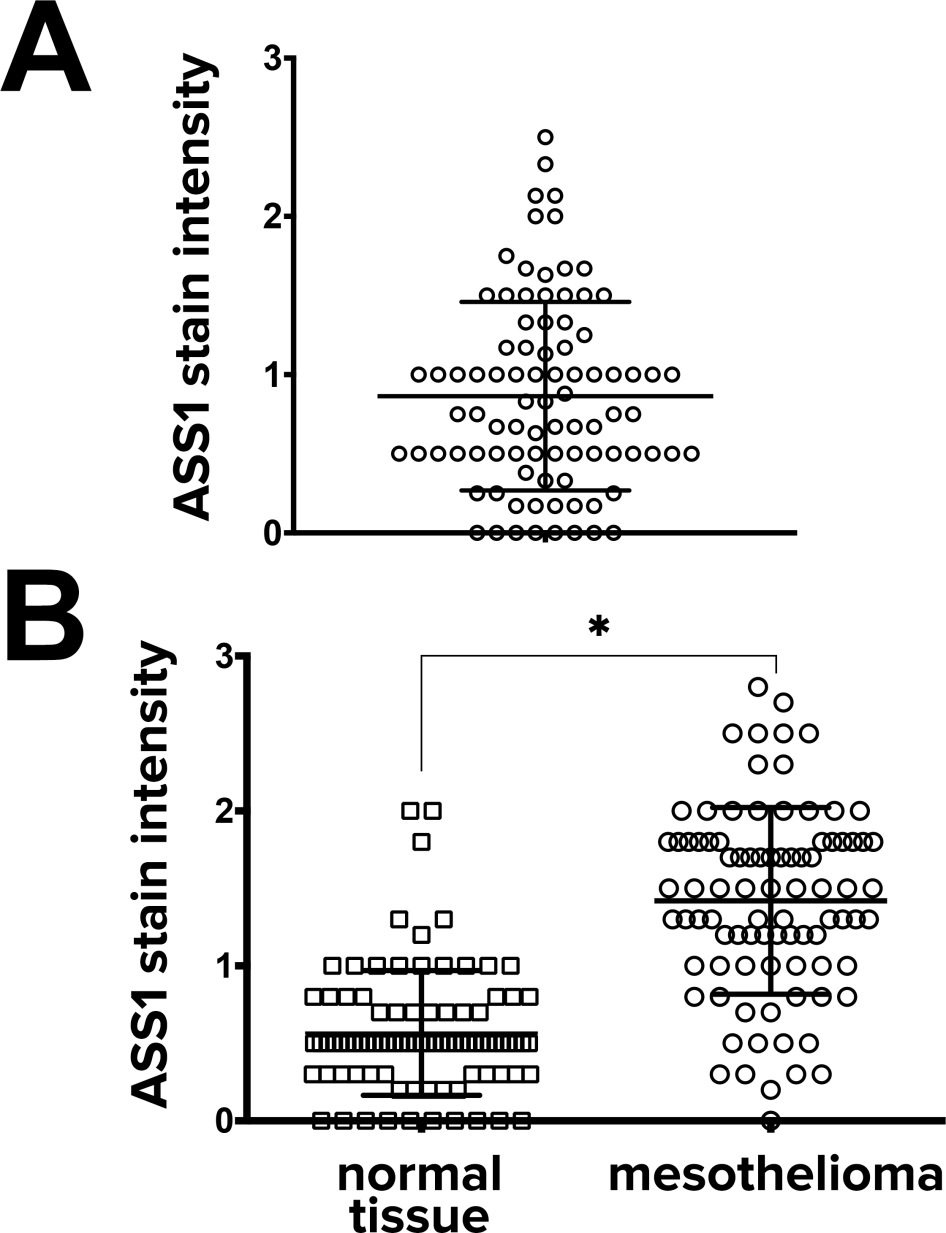
ASS1 protein is expressed in mesothelioma samples and upregulated when compared to normal tissues. **(A)** ASS1 stain intensity was measured in 88 mesothelioma samples by immunohistochemistry (each tumor in replicates of 3) in a semi-quantitative fashion as outlined in the Methods section. Only 8 samples out of 88 (10%) showed no ASS1 staining **(B)** ASS1 stain intensity was also measured in another 88 mesotheliomas with their paired 88 normal tissues. Analysis of matched pairs (tumor vs normal tissue control obtained from the same patients) of the 88 patients showed that ASS1 expression was significantly higher in mesothelioma (* *p* < 0.0001, n = 88 matched pairs).

To investigate the levels of ASS1 protein expression in human mesothelioma tumors, we performed immunohistochemistry on 2 sets of tissue microarrays (TMA) containing samples from 176 pleural mesotheliomas. In the first set of 88 mesotheliomas, only 8 mesotheliomas completely lacked ASS1 expression (Fig 4A). In another set of clinical samples, which contained 88 tumors along with their matching normal control tissue, ASS1 was significantly upregulated in mesothelioma samples compared to their matched normal controls (Fig 4B). Among the 88 mesotheliomas, there was no difference in ASS1 expression between epithelioid (n = 46) and non-epithelioid (sarcomatous and biphasic)(n = 42) mesotheliomas (S5 Table). In all 176 tumor samples, ASS1 protein was expressed in 87 (50%; more than 1+).

### ASS1 has a survival role in cells in which it is expressed

**Fig 5:**
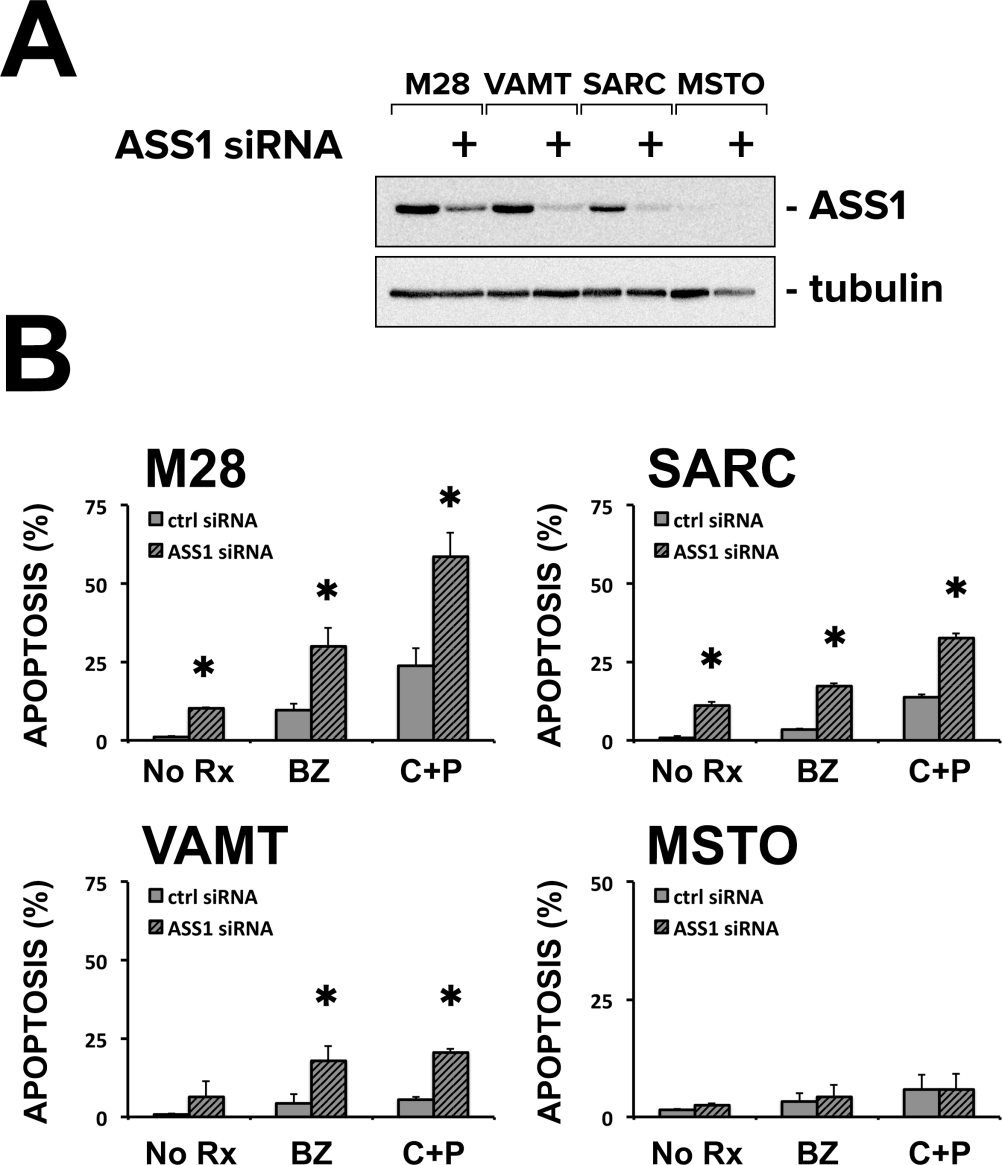
ASS1 knockdown by siRNA increases the chemosensitivity of spheroids. **(A)** Immunoblot analysis of M28, SARC, VAMT and ASS1-deficient MSTO spheroids, transfected with siRNA targeting ASS1 or control sequences show that ASS1 was efficiently knocked down in all the cells where it was expressed. **(B)** Apoptotic rates were measured for M28, SARC, VAMT and MSTO spheroids transfected with siRNA targeting ASS1 or control sequences and treated with bortezomib (BZ, 25 nM) or cis-platinum (200 μM) plus pemetrexed (10 μM)(C+P). In the 3 cell lines with baseline ASS1 expression, ASS1 siRNA increased the basal apoptotic rate and also increased the apoptotic response to chemotherapy. ASS1 siRNA had no effect in the ASS1-deficient MSTO spheroids. Apoptosis was quantified in a blinded fashion by assessing nuclear condensation in Hoechst-stained cells. (* *p* ≤ 0.05 n = 3).

The lack of ASS1 expression has been associated with resistance to platinum [24], suggesting that ASS1 could enhance chemosensitivity. On the other hand, we have found ASS1 upregulated in 3D spheroids and tumor samples and we hypothesized that it could be associated with increased chemoresistance (multicellular resistance). Thus, to clarify whether ASS1 was associated with chemosensitivity or chemoresistance, we performed RNAi experiments and measured the response to chemotherapy of spheroids in the absence of ASS1 expression. ASS1 siRNA effectively reduced ASS1 expression in 3 cell lines (M28, SARC, and VAMT) with baseline ASS1 expression (Fig 5A). In these cell lines, but not in MSTO, which do not express ASS1, reduction of ASS1 expression led to increased apoptosis. Also, in these cells, but not in ASS1-deficient MSTO, the reduction of ASS1 increased the apoptotic response to bortezomib and to cisplatin plus pemetrexed, indicating that ASS1 has survival roles in 3D and thus may contribute to chemoresistance (Fig 5B).

## Discussion

Multicellular resistance, the resistance acquired by cells in 3D, may mimic the chemoresistance of solid tumors [25]. In mesothelioma, we have investigated contributions of the PI3K/Akt pathway [26, 27] and the Bcl-2 family [28] to multicellular resistance and have found strategies to bypass such resistance mechanisms [1, 4, 5]. With this study, we asked whether a 3D gene signature could give insights into the source of multicellular resistance. Indeed, spheroids have been shown to represent the gene expression of their original tissue better than monolayers [29] and have been used to identify tumor-relevant genes associated with survival [30]. Using this screening approach, in which genes upregulated in 3D were cross-referenced against published mesothelioma genes, we found that ASS1 is upregulated in 3D and also in mesothelioma. This result was unexpected because, in the literature, mesothelioma has been described as an ASS1-deficient tumor [16–20]. Thus, there appear to be two groups of mesothelioma, those with ASS1 deficiency and those with upregulation of ASS1.

ASS1 represents the rate-limiting enzyme in the production of arginine, a semi-essential amino acid for multiple metabolic and synthetic functions [18, 31, 32]. However, in certain tumors, ASS1 has been found to be deficient [33], likely due to promoter methylation [19, 34]. Without ASS1, cells are dependent on extracellular arginine, a condition known as arginine auxotrophy. In this situation, the ASS1-deficient tumor is vulnerable to strategies that target this lifeline; accordingly, treatments to degrade extracellular arginine are being used in the patients with ASS1-deficient tumors. Such tumors have been reported to include melanoma, hepatocellular carcinoma, renal cell carcinoma, prostate cancer and mesothelioma [17]. ASS1 deficiency has also been associated with platinum resistance; in some studies, the silencing of ASS1 has induced a resistance to platinum-induced cell death while, at the same time, sensitizing to arginine degradation [24]. In the case of mesothelioma with low ASS1 expression, patients have been treated with pegylated arginine deiminase (ADI-PEG20), either alone (ADAM - https://goo.gl/hSNkI7)) or together with chemotherapy (TRAP - http://goo.gl/tiwzGO), to deprive the tumor of arginine; results from these trials are pending.

In our study, ASS1 emerged as strongly associated with the 3D phenotype, which itself is associated with chemoresistance. In addition, when tissue microarrays were examined, ASS1 protein expression was also found to be elevated in many mesothelioma tumors. In paired samples, ASS1 was elevated in tumor compared to adjacent normal tissues. Thus, ASS1 appears to be a gene relevant to mesothelioma biology, with a significance as yet unknown. Other studies have also identified the presence of ASS1 in mesothelioma; in one review of transcriptome studies, ASS1 gene was one of the most prominently increased genes in mesothelioma [9]. In addition, in the first report of ASS1 protein expression in mesothelioma, 63% of mesotheliomas were found to have low expression (0–1+) while 37% had high expression (2–3+). Our findings, if we combine all our tumors, would be 50:50; in 176 tumors, 89 demonstrate low expression (0–1+) and 87 demonstrate moderate to high (greater than 1 to 3+). Although differences can be due to different antibodies or technique, both of our studies suggest that a significant number of patients with mesothelioma will have elevated ASS1 expression. Thus, while ASS1 deficiency is undoubtedly of interest in this tumor, ASS1 overexpression also merits attention.

The role of ASS1 in tumor biology has been confusing, with ASS1 thought to act either as a tumor suppressor [35] or as pro-metastatic or carcinogenic [36]. We propose that the function of ASS1, a gene whose expression increases in 3D, may better be studied in 3D. In our 3D spheroids, ASS1 showed a survival role. The reduction of ASS1 protein by siRNA led to an increased sensitivity to chemotherapy, including to cis-platinum. We speculate that ASS1 upregulation is in response to an increased need or decreased supply of arginine in 3D and in the tumor and would be a logical step in promoting survival of the tumor cells. If so, then techniques to suppress ASS1 could improve response to chemotherapy and would also render the tumor vulnerable to arginine depletion techniques [23], such as ADI-PEG20. Further studies are underway to identify small molecules or other strategies to inhibit ASS1 both to understand its function in mesothelioma and to provide possible therapeutic options to patients with high ASS1 expression.

## Methods

### Cell culture and reagents

The human mesothelioma cell lines M28 [37] and VAMT [38] (both from Dr. Brenda Gerwin, NCI, National Institutes of Health, Bethesda, MD, USA), REN [39] (from Dr. Roy Smythe, University of Texas M.D. Anderson Cancer Center, Houston, TX, USA), SARC [40] (MesoSA1 –from Dr. Alice Boylan, Medical University of South Carolina, Charleston, MC, USA), JMN [41] and MSTO-211H [ATCC^®^ CRL-2081^™^] (both from Dr. Dean Fennell, University of Leicester, UK), MSTO pIRES-EV and MSTO pIRES-ASS1 [24] (both kindly donated by Dr. Peter Szlosarek, Barts Cancer Institute, Queen Mary University of London, UK), were all cultured in Dulbecco’s modified Eagle’s medium (DMEM) supplemented with 10% fetal bovine serum in a 37°C humidified incubator with 5% CO_2_. All were found to stain positively for mesothelioma markers (calretinin, WT1) and negatively for other markers not seen in mesothelioma (TTF1). Normal human mesothelial cells were cultured from ascites fluid from unidentified patients without infection or malignancy according to a protocol (#12–08998) approved by the University of California, San Francisco Committee on Human Research under our BUA (BU031309-03) [42]. All cells were confirmed to be negative for mycoplasma every 2 months by PCR analysis as previously described [43]. Bortezomib was from Selleck Chem (Boston, MA, USA). Cisplatin and pemetrexed were obtained from the University of California, San Francisco Pharmacy at Mt. Zion Cancer Center.

### Generation and treatment of spheroids

Multicellular spheroids were generated in non-adsorbent round-bottomed 96-well plates coated with a 1:24 dilution of polyHEMA (#P3932 Sigma-Aldrich, St. Louis, MO, USA) in 95% ethanol and dried at 37°C for 48 h. Plates were sterilized with UV-light for 30 min before use. In each well, 10,000 cells were added and plates were centrifuged at 1000 rpm for 5 min to concentrate the cells at the bottom and incubated for 24 h to allow formation into a solid spheroid. Before treatment, 24 multicellular spheroids were transferred to each well of a polyHEMA-coated 24-well and treated for 24 h unless stated otherwise.

### Microarray

Sample preparation, RNA extraction/amplification, labelling, and array hybridizations were performed according to standard protocols from the UCSF Shared Microarray Core Facilities ([44], http://arrays.ucsf.edu/protocols). A total of 18 samples (three for monolayers and three for spheroids for each cell line) were analyzed. Labeled cRNAs were hybridized to 70-mer oligonucleotides microarrays produced using the Operon Human Genome 70-mer Oligo Set Version 2.0 (Operon Biotechnologies, Huntsville, AL). Additional information about microarray protocols and the complete array data are available from Gene Expression Omnibus (http://www.ncbi.nlm.nih.gov/geo/, series accession GSE4804). Expression profiling of monolayers and spheroids for M28, REN and VAMT mesothelioma cells was carried out using three biological replicates in each cell type and two technical replicates (Cy3/Cy5 dye-swap replicates) for each sample. The data were normalized using robust locally weighted regression (loess) to correct for intensity, spatial and other dye biases [45, 46]. The differential expression of genes in spheroid with respect to monolayer was analyzed using the BioConductor LIMMA package [http://www.bioconductor.org]. We estimated the mean log-ratios and calculated moderated t-statistic, B statistic, false discovery rate and p-value for each gene for the direct comparisons between monolayers and spheroids. P values were adjusted for multiple testing using Bonferroni correction. In order to have the broadest selection of genes to compare the 3 cell types, we selected candidate differentially-expressed genes using adjP < 0.05 without cutoffs on fold-change. Genes were considered upregulated when the log2 ratio (M value) was > 0 (i.e. fold-change between the conditions of interest > 1); genes were considered downregulated when the log2 ratio < 0 (i.e. fold-change < 1).

### Gene Ontology analysis

The Ingenuity Interactive Pathway Analysis of Complex ‘Omics Data platform was used to investigate the functional associations between differentially expressed genes. Analysis was performed by the CNRS and University Nice Sophia Antipolis, IPMC, Valbonne, France. We also interrogated the Database for Annotation, Visualization and Integrated Discovery (DAVID) functional annotation tool (http://david.abcc.ncifcrf.gov) by using the full list of differentially expressed genes in 3D.

### Real-Time qRT-PCR

RNA was extracted from monolayers (1 million cells) or spheroids (100 spheroids) with an RNeasy Kit following manufacturer’s instructions (Qiagen, Valencia, CA, USA). Total RNA was quantified with a NanoDrop 2000c (NanoDrop, Wilmington, DE, USA) and 2 μg of total RNA was retro-transcribed with a Superscript VILO cDNA Synthesis Kit (Life Technologies, Carlsbad, CA, USA). A TaqMan Gene Expression Analysis Assay (Life Technologies, Carlsbad, CA, USA) was performed according to manufacturer’s instructions in an ABI7000 thermal cycler (Life Technologies, Carlsbad, CA, USA). TaqMan probes for ASS1 (Hs01597989_g1), ANXA4 (Hs00984874_m1), MVP (Hs00245438_m1) and the control beta glucuronidase (GUSB, 4333767T) were from Applied Biosystems (Life Technologies, Carlsbad, CA, USA). Calculations for determining the relative levels of gene expression were made from triplicate or quadruplicate measurements of the target gene, with normalization to GUSB in the samples, using the cycle threshold (Ct) method and the 2^-ddCT equation [47].

### SDS-PAGE and immunoblotting

Monolayers and spheroids were lysed in boiling lysis buffer (2.5% SDS, Tris-HCl 250 mM pH 7.4) and protein concentration was determined using a colorimetric assay (DC protein assay from BioRad, Hercules, CA, USA). 30 μg of protein was supplemented with Laemmli sample buffer and separated on an SDS-PAGE gel. Proteins were transferred to PVDF membranes (Immobilon, Millipore, Billerica, MA, USA) and blocked with 5% Blotto (Santa Cruz Biotechnology, Dallas, TX, USA) in PBS. Primary antibodies were diluted in 5% non-fat dry milk or 5% BSA. The following primary antibodies were used: anti-ASS1 (clone 25, 1:500, BD Biosciences, San Jose, CA, USA; clone 2B10, 1:500, Sigma-Aldrich, St. Louis, MO, USA; clone 2C10, 1:500, Millipore, Billerica, MA, USA), anti-α-tubulin (#A5441, 1:10,000, Sigma-Aldrich, St. Louis, MO, USA). Secondary antibodies were from BioRad (Hercules, CA, USA). Chemiluminescence was detected with the enhanced SuperSignal West Pico Substrate (Pierce, Rockford, IL, USA) with a Biospectrum Imaging System (UVP, Upland, CA, USA).

### Human subjects research

The University of California San Francisco CHR approved the studies on human samples under an exempt category (#12–08998). CHR protocol 12–08998 was reviewed by the University of California San Francisco Committee on Human Research, Laurel Heights Committee (CHR FWA number: 00000068; IRB registration number: 00003471). Exemption was granted because the research involves the collection or study of existing data, documents, records, pathological specimens, or diagnostic specimens or because the information is recorded by the investigator in such a manner that subjects cannot be identified, directly or through identifiers linked to the subjects. Both of these statements were met in our research. Moreover, the coded private information or specimens were not collected specifically for the current proposed research project and the key to decipher the code is destroyed before researcher begins. Samples were obtained from surgeries performed by Dr. Raphael Bueno, Chief, Division of Thoracic Surgery at Brigham and Women's Hospital, Boston MA with consent from Dana Farber/Harvard Cancer Center IRB-approved protocol 98–063. The University of California San Francisco CHR committee approved the use of these unidentified mesothelioma samples for our research. Normal mesothelial cells were obtained under the same CHR-exempt authorization from unidentified patients' material (ascites or pleural fluid) that would otherwise be discarded. Clinical tumor micro arrays, obtained under the same CHR protocol (12–08998), were created by the pathology core facilities of the Dana Farber/Harvard Cancer Center, with coded links to patient identity under protocol 98–063

### Immunohistochemistry

We utilized 2 sets of tumor microarrays (TMA): one set contained 88 chemonaïve mesothelioma samples (created by the Pathology Core facility at University of California San Francisco, Mt. Zion with anonymous mesothelioma samples obtained from our collaboration with Drs. Bueno and Richards) and one set contained a different 88 mesotheliomas with matching normal tissue, created from clinical pathology blocks of Brigham and Women's Hospital patients. In addition to anonymous control cores, microarray blocks contained, per included patient, 3 cores of tumor and, if available, 3 cores of normal tissue obtained from selected areas of archival clinical blocks. Sections of TMAs (5 μm) were stained for 2 h at RT with antibodies for ASS1 (clone 2C10, 1:1000, Millipore, Billerica, MA, USA) and visualized with a HRP/DAB Envision plus Kit (#K4010, Dako, Carpinteria, CA). Known negative and positive controls were included. Hematoxylin was used as a counterstain. Slides were viewed by two independent scorers and scored semi-quantitatively (0 = no staining, 1 = weak staining, 2 = moderate staining, 3 = strong staining) [18].

### Nuclear condensation assay by Hoechst staining

Spheroids were disaggregated with trypsin, washed with ice-cold PBS, and then fixed with 2.5% glutaraldehyde (Sigma-Aldrich, St. Louis, MO, USA). Cells were then stained with 8 mg/ml of Hoechst 33342 (Molecular Probes, Life Technologies, Carlsbad, CA, USA) and placed on slides. Apoptosis was quantitated by counting cells with distinctive signs of nuclear condensation and expressed as a % of the total cells. For each condition, at least 300 cells were counted in triplicate by investigators blinded to the experimental conditions. We have found that this assay is more accurate than assays that detect surface phosphatidylserine in cells disaggregated from spheroids [1].

### RNA interference

Transient knockdowns were performed using Lipofectamine RNAiMAX according to the manufacturer’s protocol (Life Technologies, Carlsbad, CA, USA). In brief, cells were transfected in Opti-MEM (Life Technologies, Carlsbad, CA, USA) and allowed to grow as monolayers for 24 h in complete DMEM medium. Cells were then trypsinized, counted, plated in polyHEMA plates and allowed to form spheroids for 24 h. Spheroids were then treated as described in figure legends, between 48 and 72 h from transfection. The human argininosuccinate synthetase 1 (ASS1) siRNA (Stealth HSS181354, 5’-AGC AGC UGA GCU CAA ACC GGA CCU-3’) and the Stealth siRNA Negative Control lo GC (#12935) were purchased from Life Technologies, Carlsbad, CA, USA.

### Statistical analysis

Data are expressed as mean ± standard deviation of at least three different experiments. Statistical significance was evaluated by ANOVA (with Tukey’s test to determine where the difference lay). Differences between the tumor and normal tissue matched pairs and between epithelioid and non-epithelioid tumors were evaluated by the Mann-Whitney non-parametric test. A *p* value *≤* 0.05 was considered significant. (GraphPad Prism v 4.0, GraphPad Software, Inc., La Jolla, CA, USA).

## Supporting Information

S1 File Raw data for Figs 2, 4A and 4B and 5B. (ZIP)

S2 File Unedited Western Blots. (ZIP)

S1 Table Upregulated genes in 3D. The table shows the 138 genes upregulated in 3D spheroids grown from M28, REN and VAMT cell lines.
(PDF)

S2 Table Downregulated genes in 3D. The table shows the 71 genes downregulated in 3D spheroids grown from M28, REN and VAMT cell lines.
(PDF)

S3 Table Ingenuity^®^ analysis of the genes differentially expressed by mesothelioma spheroids compared to respective monolayers. The table shows the top functions altered in 3D in the signaling families: *molecular and cellular functions*, *physiological system development and function*, and *diseases and disorders*. The pathways affected in the different categories are shown in the table, together with p value and number of differentially expressed genes in each pathway (*Count*). A comprehensive analysis of the functions affected by the differentially-expressed genes did not reveal a single suppressed or activated pathway.
(PDF)

S4 Table DAVID Analysis of the genes differentially expressed in 3D. The table shows the DAVID functional annotation chart with *p*<0.01 for the differentially expressed genes in 3D.
(PDF)

S5 Table ASS1 expression is not statistically different between epithelioid and non-epithelioid mesotheliomas. The table shows the ASS1 staining intensity of 88 mesotheliomas, categorized by their histotypes: epithelioid (46) and non epithelioid (42—sarcomatous and biphasic). The difference between the two groups was not statistically significant (*p* = 0.5662).
(PDF)

## Abbreviations

2D: Two-dimensional
3D: three-dimensional
MCS: multicellular spheroids
TFS: tumor fragment spheroids
BZ: bortezomib
C+P: cisplatin plus pemetrexed
ASS1: argininosuccinate synthase 1
MVP: major vault protein
ANXA4: annexin A4
RNAi: RNA interference
siRNA: small interfering RNA
