# Supplementary figures and images for: Analysis of Gene Expression in 3D Spheroids Highlights a Survival Role for ASS1 in Mesothelioma
Source: PLoS One. 2016 Mar 16;11(3):e0150044. doi: 10.1371/journal.pone.0150044 (PMC4794185; doi:10.1371/journal.pone.0150044)

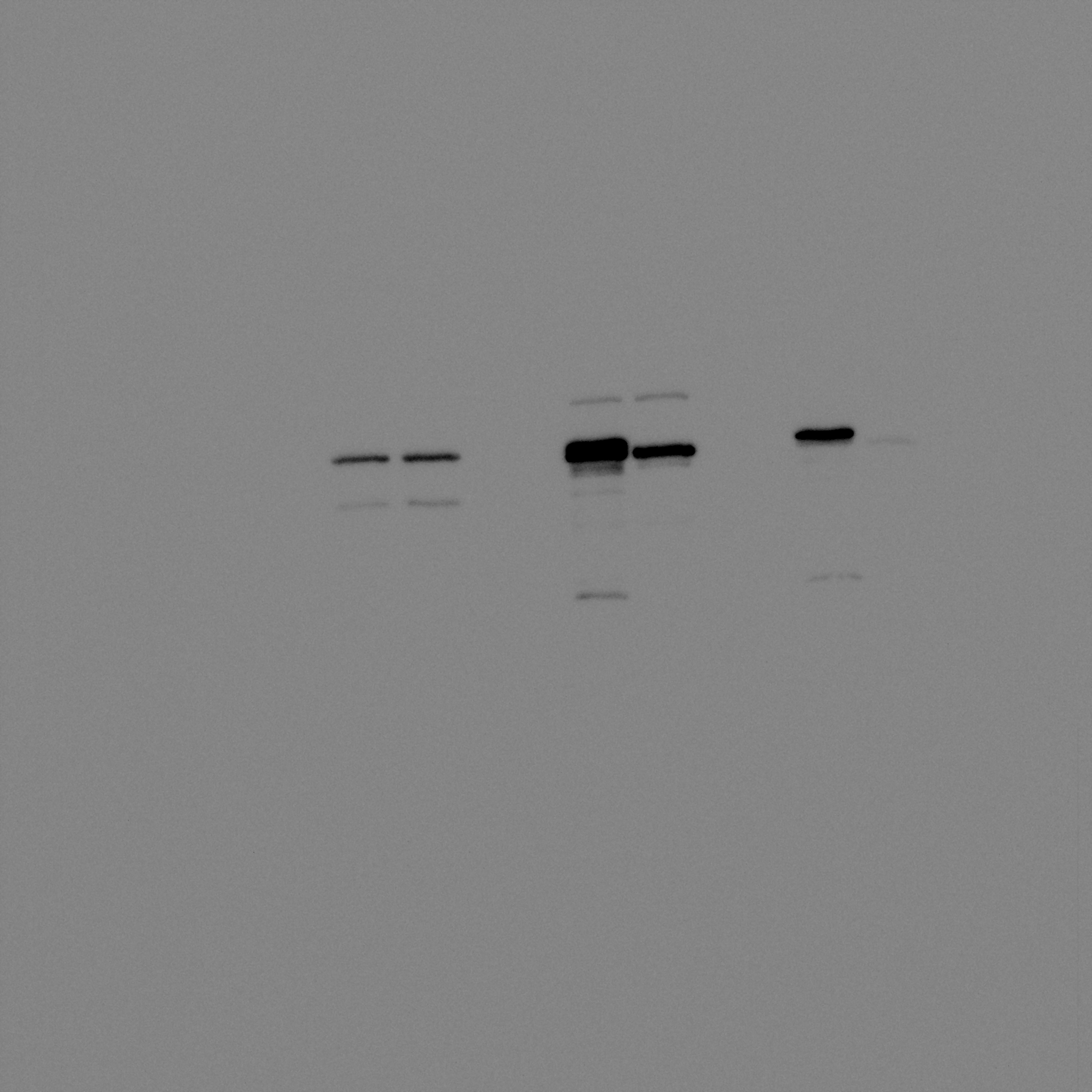

Supplement: S2 File — (ZIP) [file pone.0150044.s002.zip › FIGURE 3A/Figure 3A_ASS1.tiff]

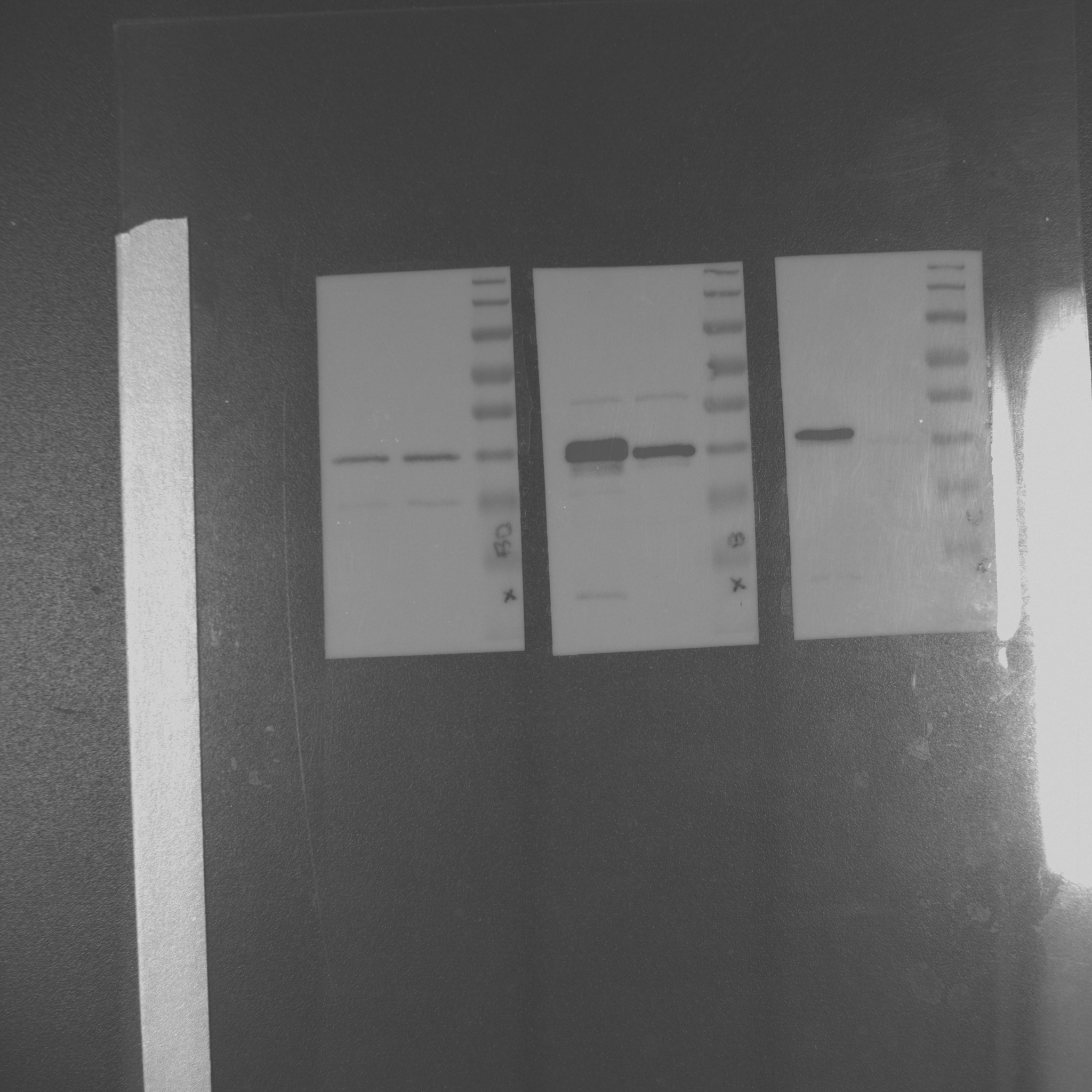

Supplement: S2 File — (ZIP) [file pone.0150044.s002.zip › FIGURE 3A/Figure 3A_ASS1_MW.tiff]

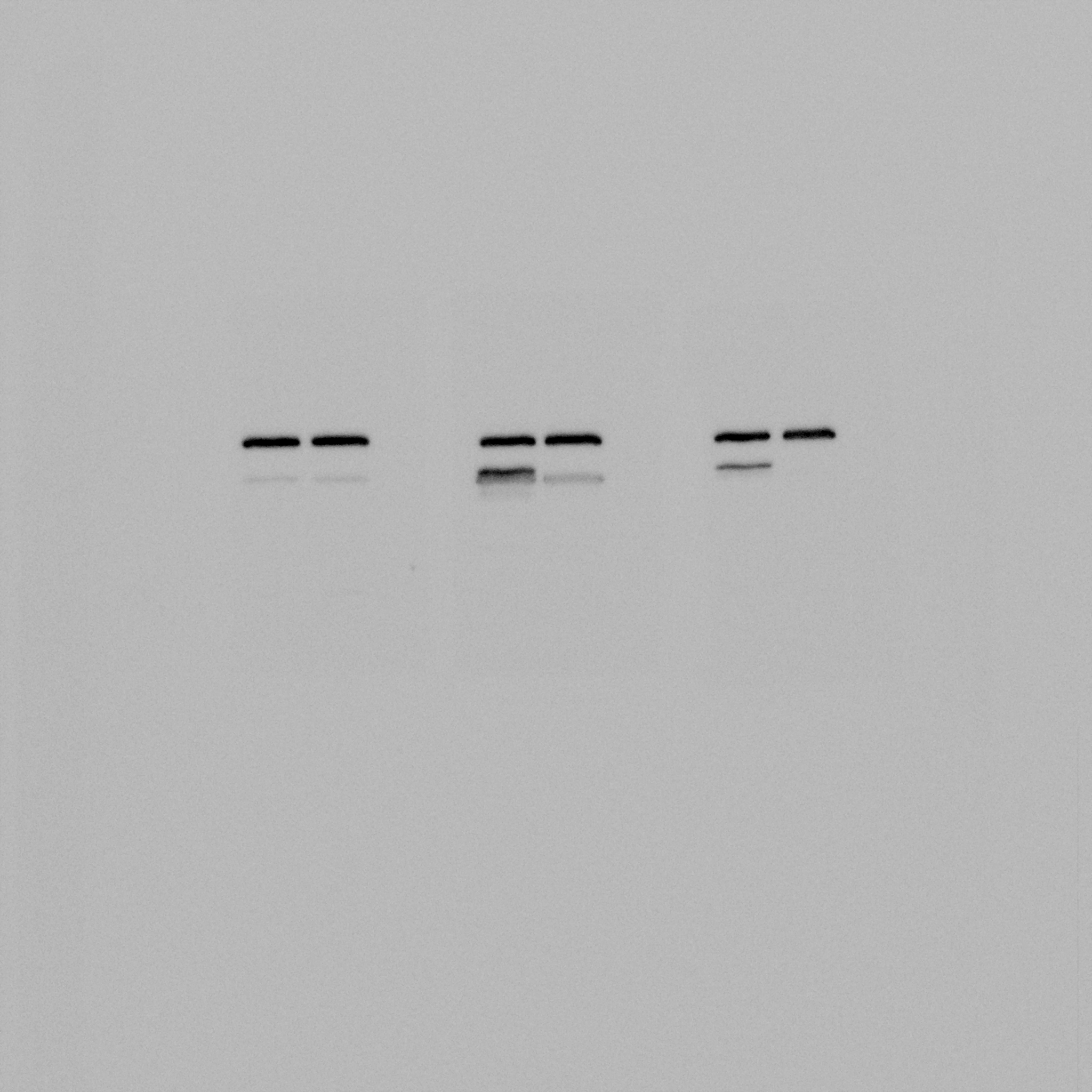

Supplement: S2 File — (ZIP) [file pone.0150044.s002.zip › FIGURE 3A/Figure 3A_tubulin.tiff]

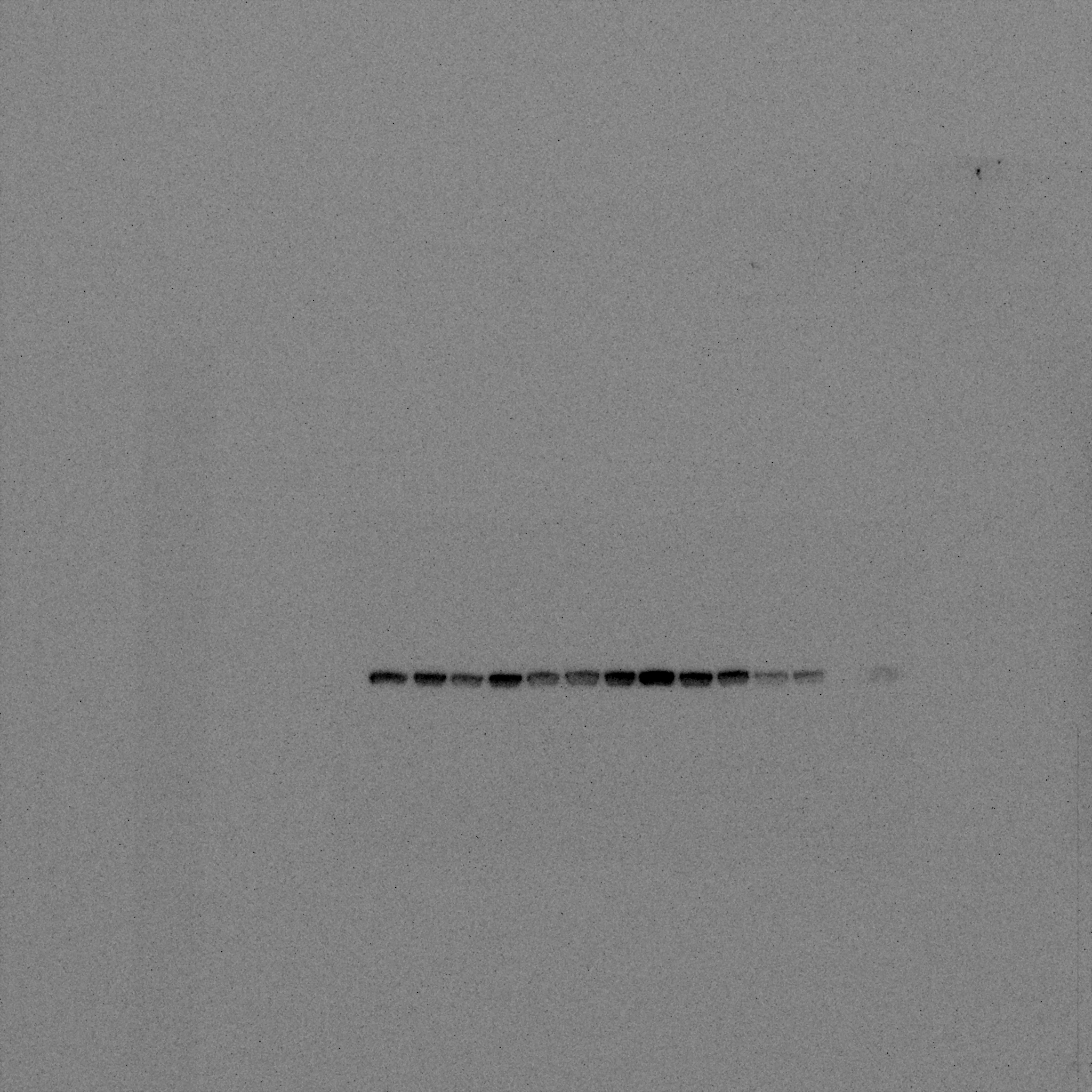

Supplement: S2 File — (ZIP) [file pone.0150044.s002.zip › FIGURE 3B/Figure 3B_ANXA4.tiff]

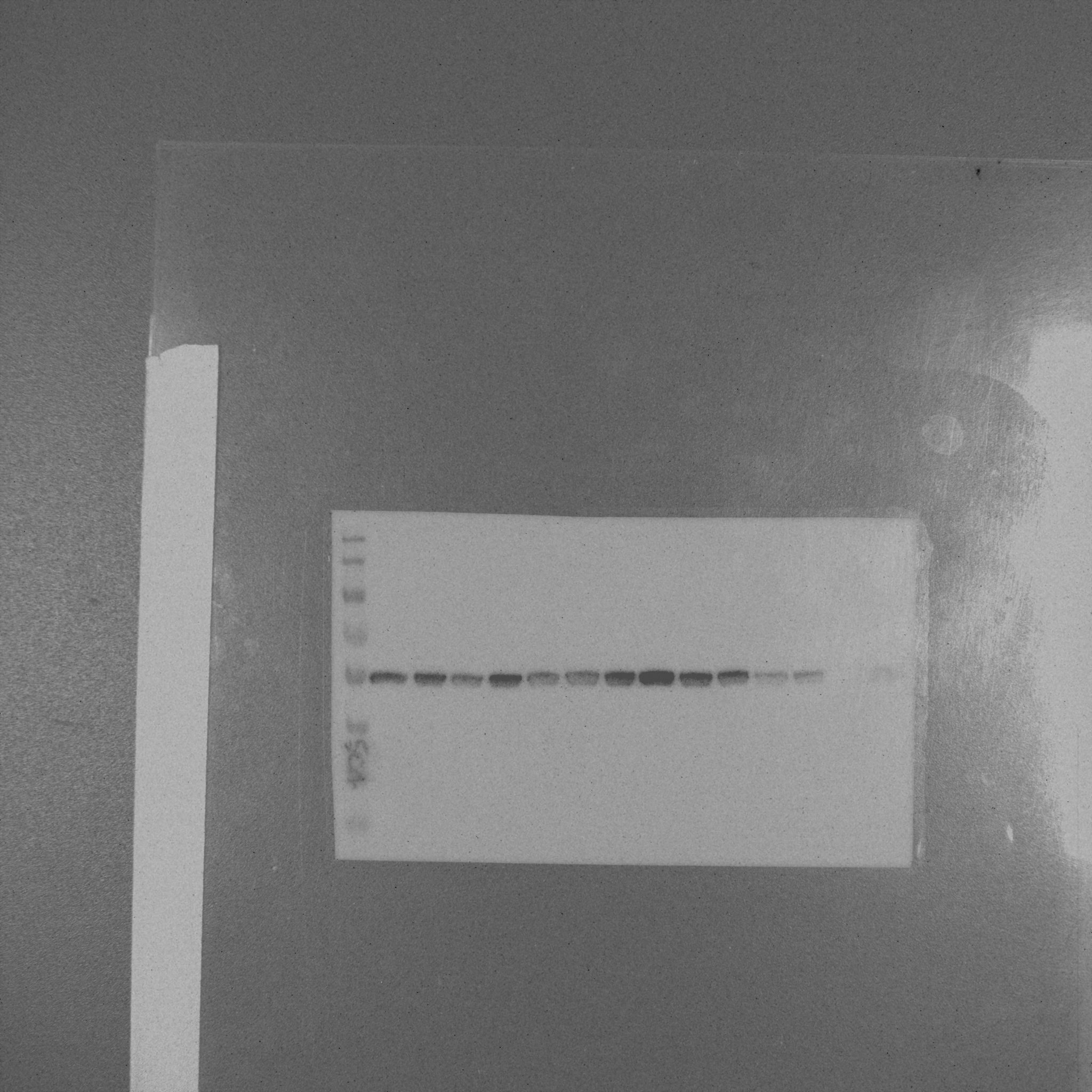

Supplement: S2 File — (ZIP) [file pone.0150044.s002.zip › FIGURE 3B/Figure 3B_ANXA4_MW.tif]

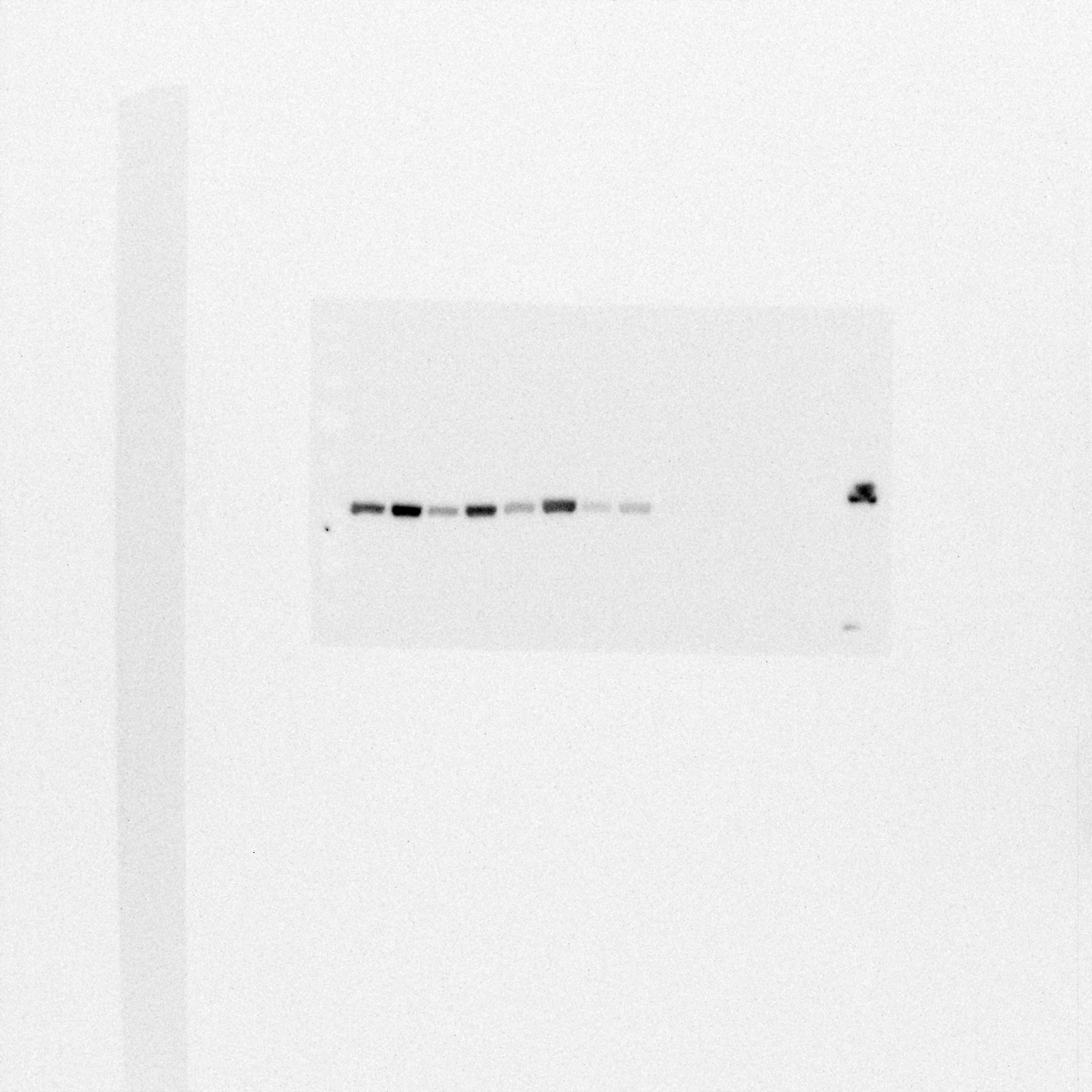

Supplement: S2 File — (ZIP) [file pone.0150044.s002.zip › FIGURE 3B/Figure 3B_ASS1.tiff]

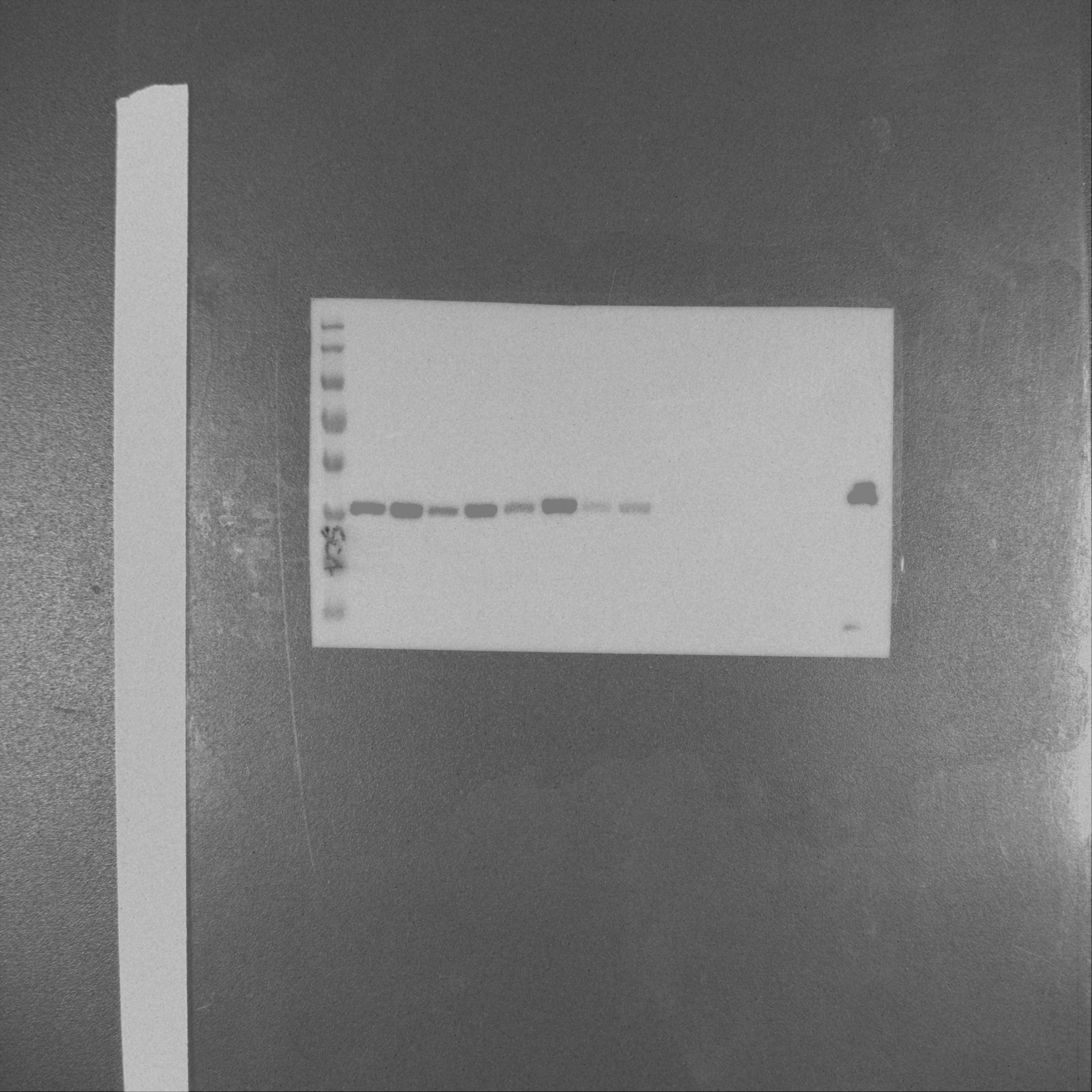

Supplement: S2 File — (ZIP) [file pone.0150044.s002.zip › FIGURE 3B/Figure 3B_ASS1_MW.tiff]

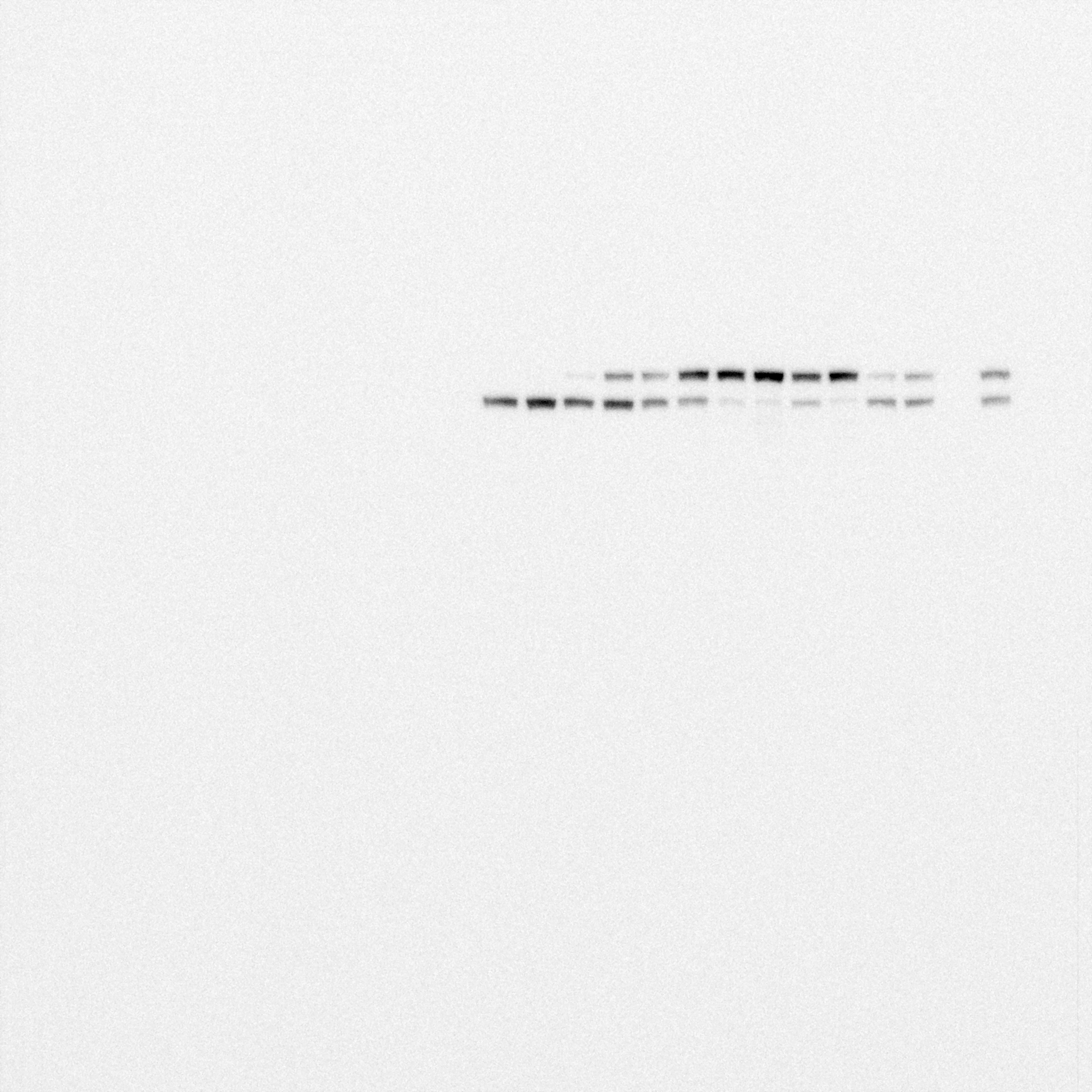

Supplement: S2 File — (ZIP) [file pone.0150044.s002.zip › FIGURE 3B/Figure 3B_MVP.tiff]

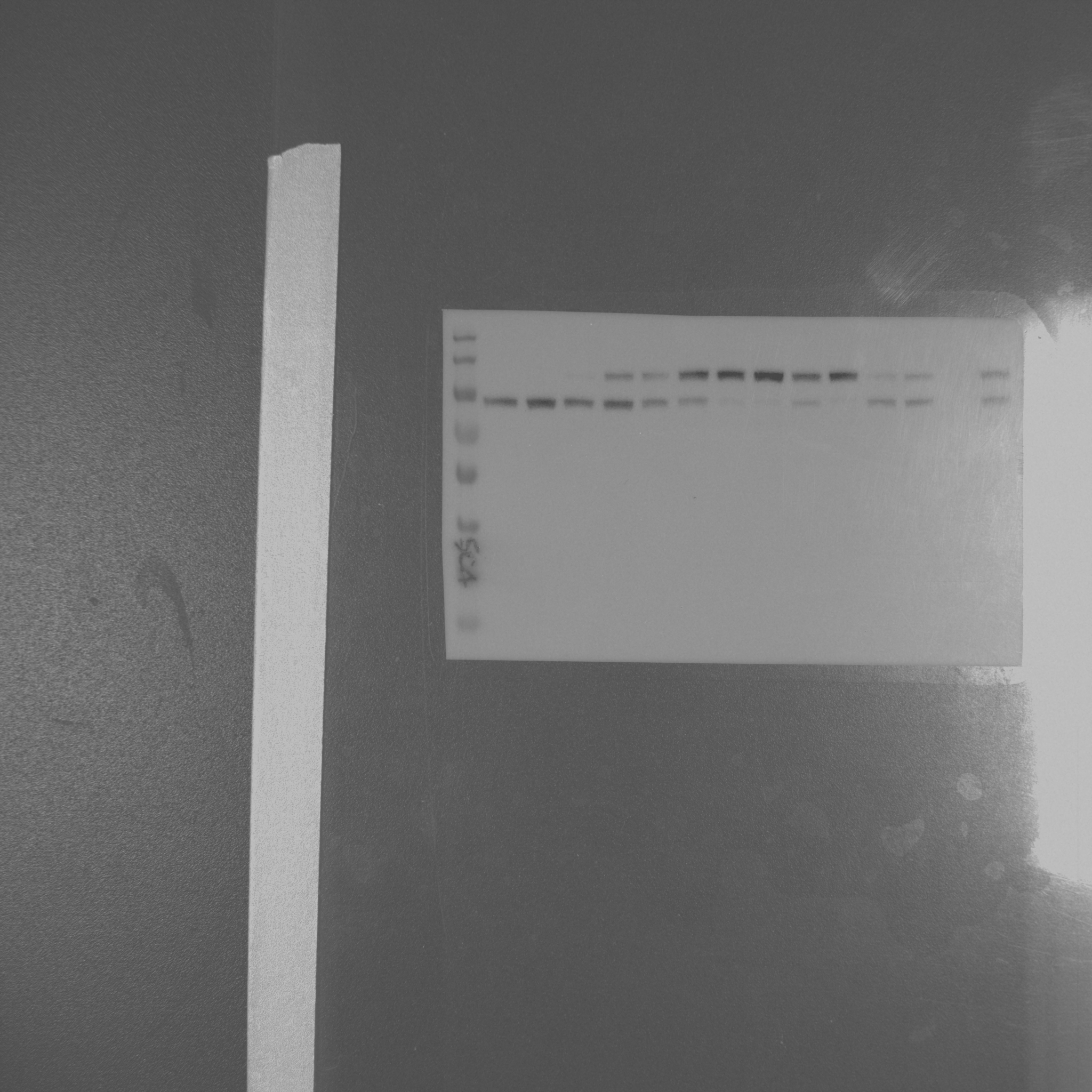

Supplement: S2 File — (ZIP) [file pone.0150044.s002.zip › FIGURE 3B/Figure 3B_MVP_MW.tiff]

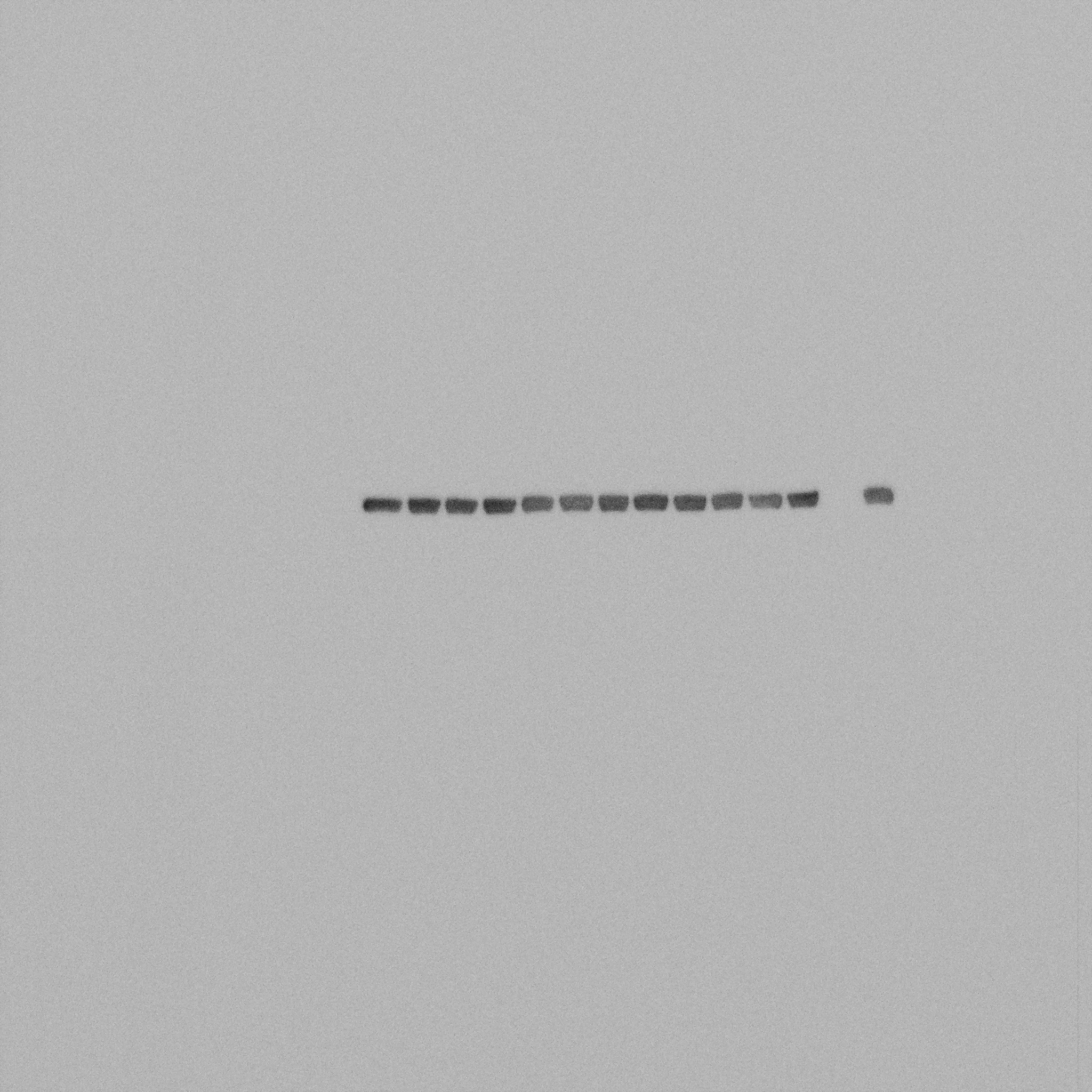

Supplement: S2 File — (ZIP) [file pone.0150044.s002.zip › FIGURE 3B/Figure 3B_tubulin.tiff]

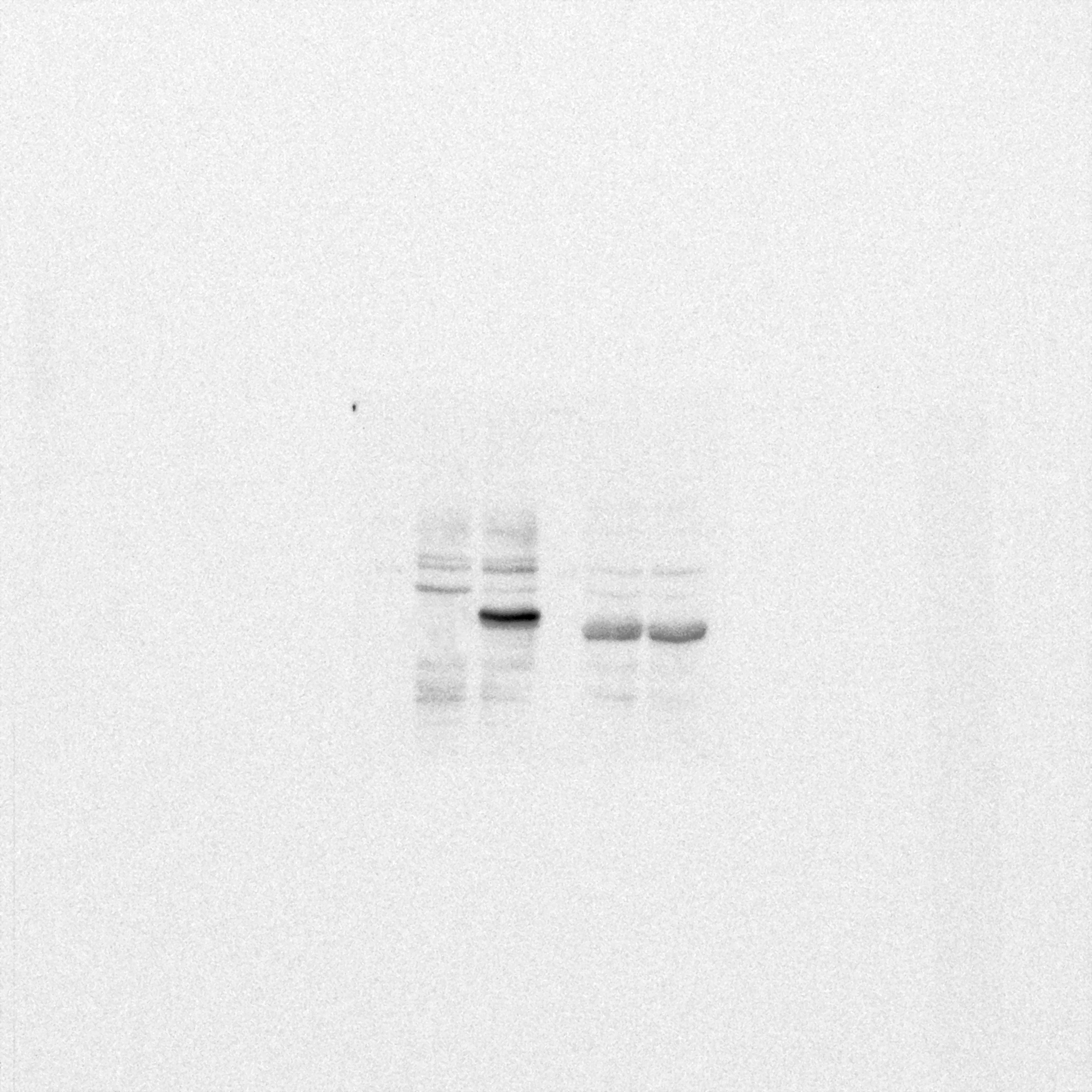

Supplement: S2 File — (ZIP) [file pone.0150044.s002.zip › FIGURE 3C/Figure 3C_ASS1.tiff]

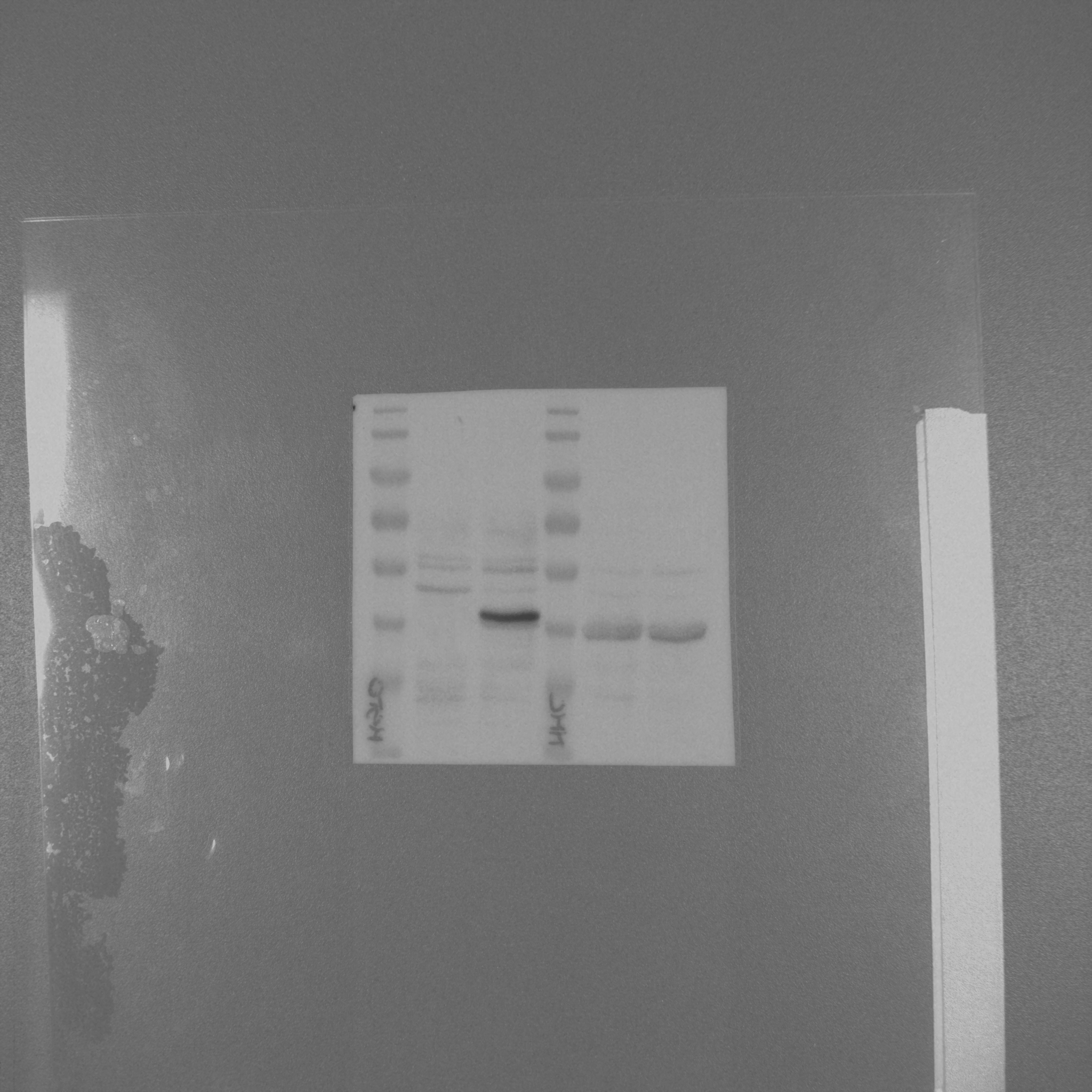

Supplement: S2 File — (ZIP) [file pone.0150044.s002.zip › FIGURE 3C/Figure 3C_ASS1_MW.tiff]

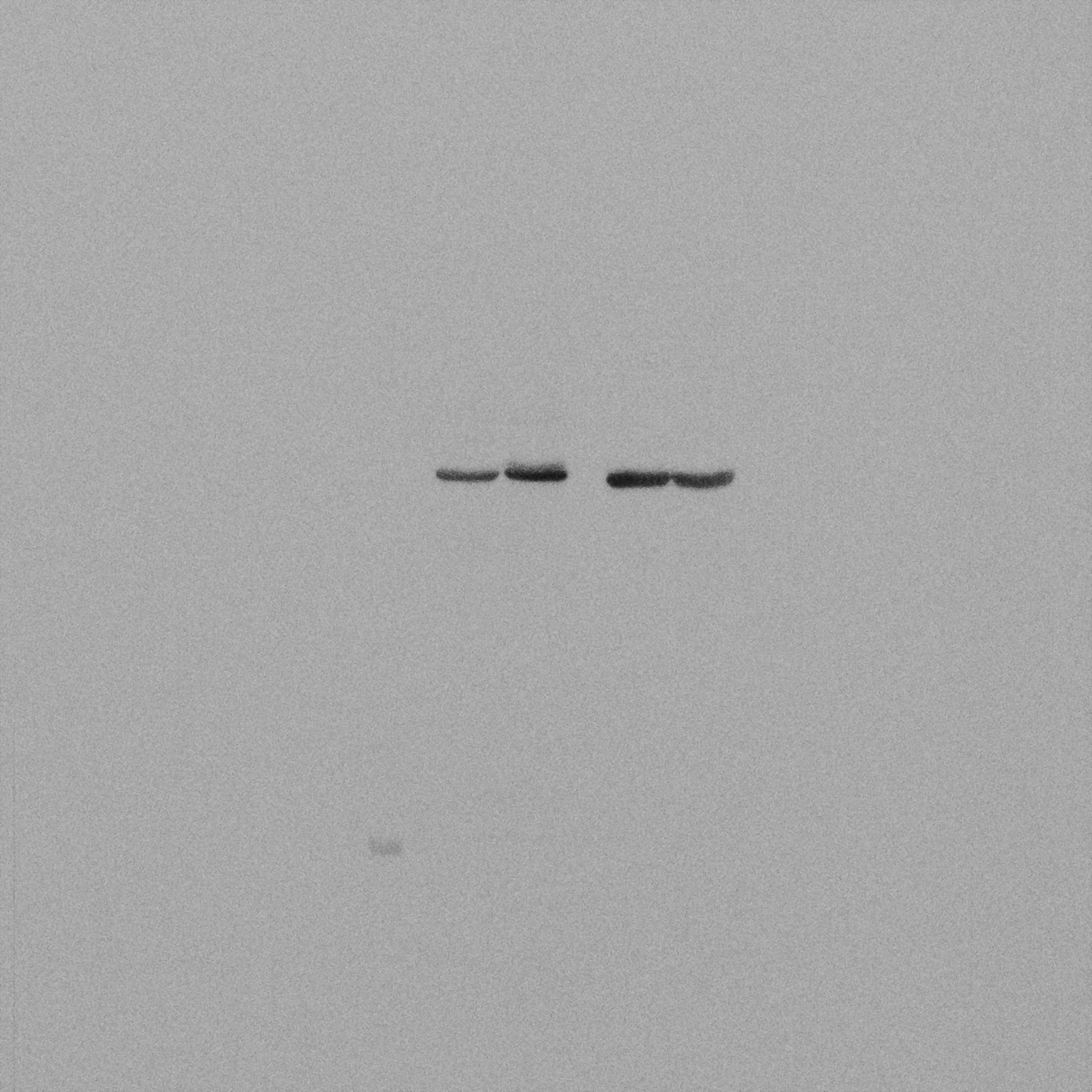

Supplement: S2 File — (ZIP) [file pone.0150044.s002.zip › FIGURE 3C/Figure 3C_tubulin.tiff]

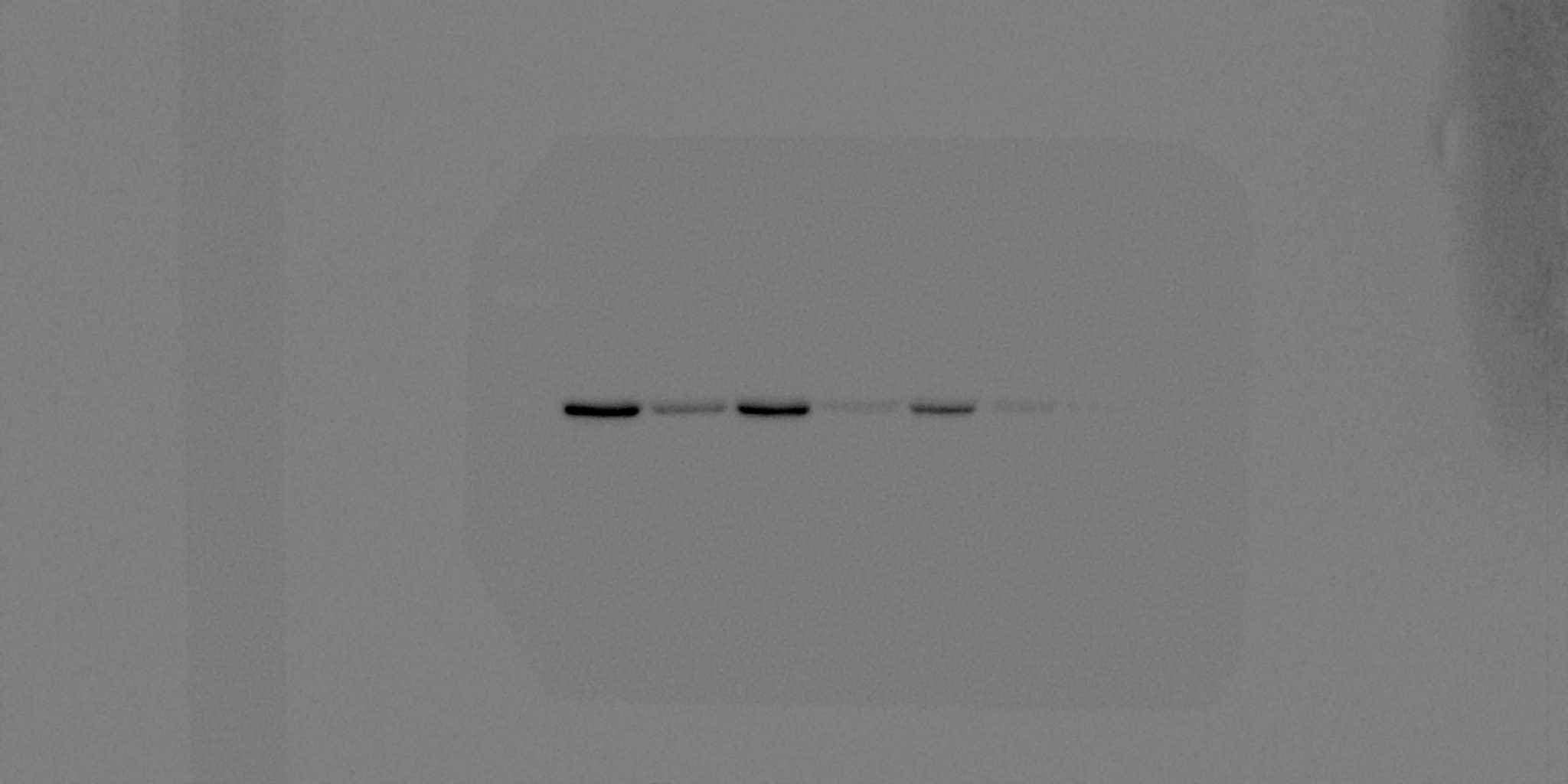

Supplement: S2 File — (ZIP) [file pone.0150044.s002.zip › FIGURE 5A/Figure 5A_ASS1.tiff]

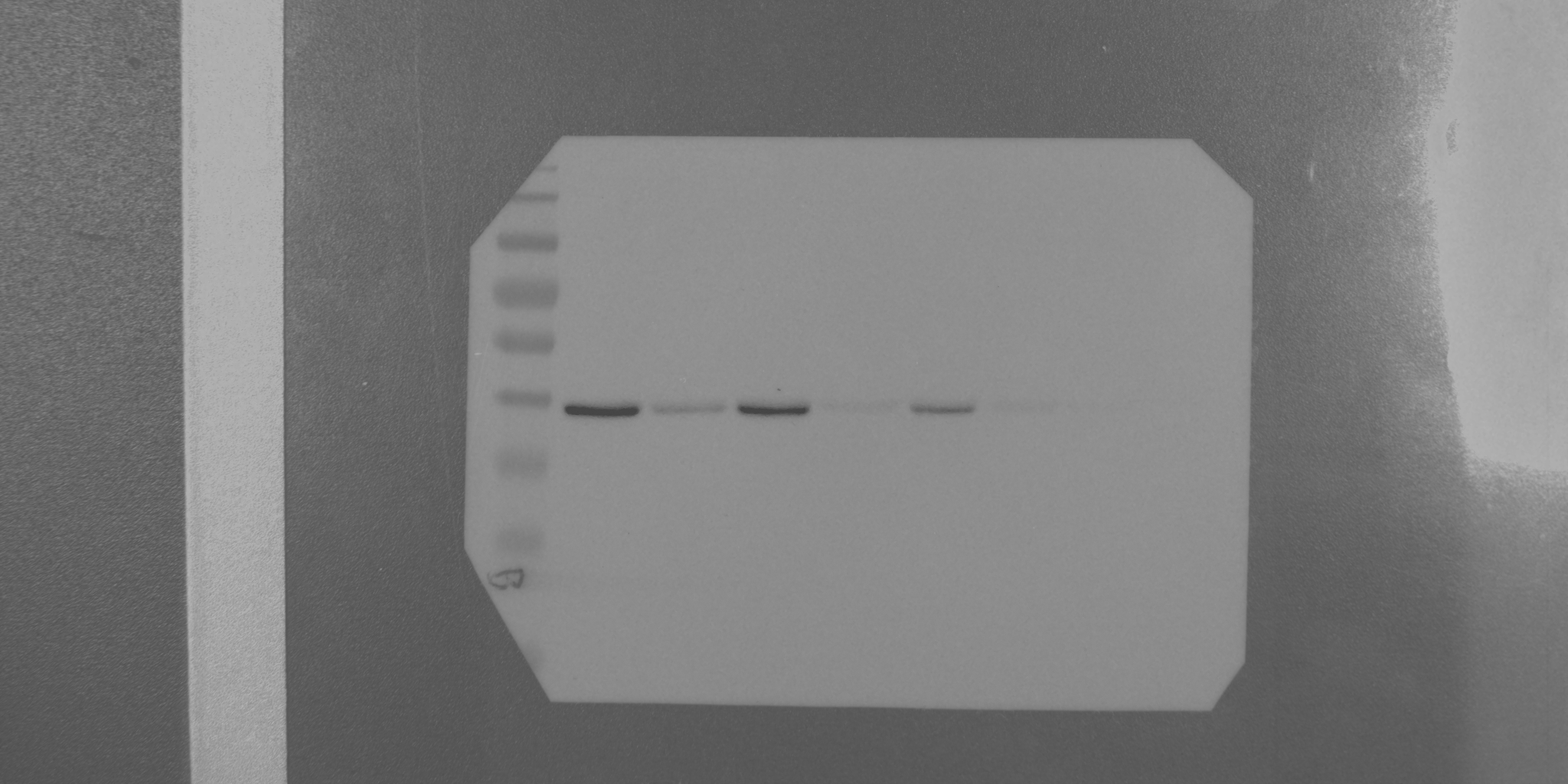

Supplement: S2 File — (ZIP) [file pone.0150044.s002.zip › FIGURE 5A/Figure 5A_ASS1_MW.tiff]

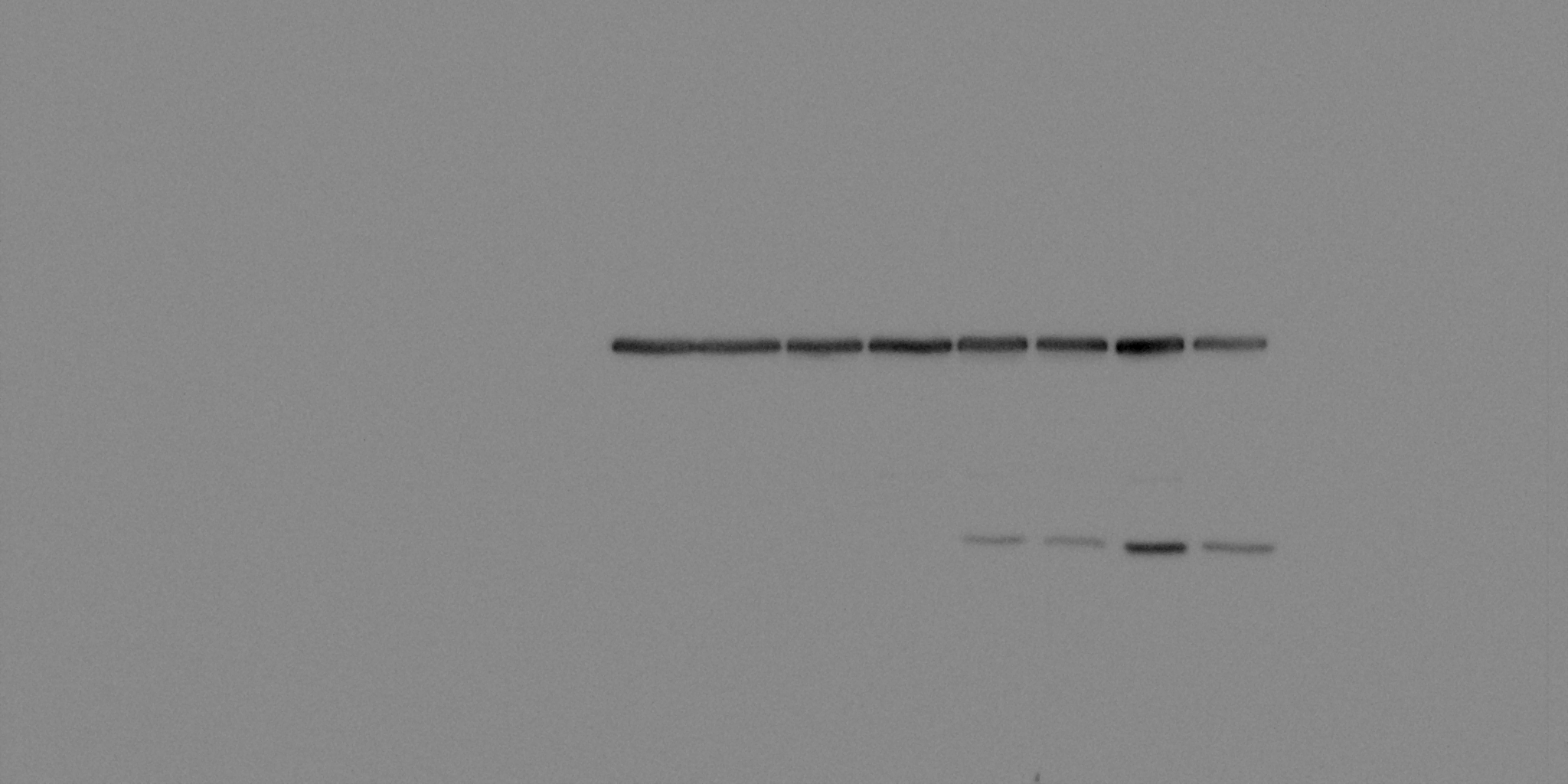

Supplement: S2 File — (ZIP) [file pone.0150044.s002.zip › FIGURE 5A/Figure 5A_tubulin.tiff]
