# Supplementary material for: Analysis of Gene Expression in 3D Spheroids Highlights a Survival Role for ASS1 in Mesothelioma
Source: PLoS One. 2016 Mar 16;11(3):e0150044. doi: 10.1371/journal.pone.0150044 (PMC4794185; doi:10.1371/journal.pone.0150044)
Supplement: S1 Table — The table shows the 138 genes upregulated in 3D spheroids grown from M28, REN and VAMT cell lines. (PDF) [file pone.0150044.s003.pdf]

Table S1 | **Upregulated genes in mesothelioma spheroids**

Gene Symbol	Description	Unigene ID
ABCG2	ATP-binding cassette, sub-family G (WHITE), member 2	Hs.480218
ADD3	adducin 3 (gamma)	Hs.501012
AKR1B1	aldo-keto reductase family 1, member B1 (aldose reductase)	Hs.521212
AKR1B10	aldo-keto reductase family 1, member B10 (aldose reductase)	Hs.116724
AKR1C1	aldo-keto reductase family 1, member C1 (dihydrodiol dehydrogenase 1; 20-alpha (3-alpha)-hydroxysteroid dehydrogenase)	Hs.460260
ANXA4	annexin A4	Hs.422986
APOBEC3C	apolipoprotein B mRNA editing enzyme, catalytic polypeptide-like 3C	Hs.441124
APOBEC3G	apolipoprotein B mRNA editing enzyme, catalytic polypeptide-like 3G	Hs.474853
APOL1	apolipoprotein L, 1	Hs.114309
APOL6	apolipoprotein L, 6	Hs.257352
AQP3	aquaporin 3 (Gill blood group)	Hs.234642
ARL6IP5	ADP-ribosylation-like factor 6 interacting protein 5	Hs.518060
ASS1	argininosuccinate synthetase 1	Hs.160786
ATF3	activating transcription factor 3	Hs.460
ATXN1	ataxin 1	Hs.434961
B2M	beta-2-microglobulin	Hs.48516
BST2	bone marrow stromal cell antigen 2	Hs.118110
BTN3A1	butyrophilin, subfamily 3, member A1	Hs.191510
BTN3A3	butyrophilin, subfamily 3, member A3	Hs.167741
C10orf39	chromosome 10 open reading frame 39	Hs.106254
C15orf48	chromosome 15 open reading frame 48	Hs.112242
C16orf45	chromosome 16 open reading frame 45	Hs.460095
C1R	complement component 1, r subcomponent	Hs.376414
C3orf34	chromosome 3 open reading frame 34	Hs.334526
CAPG	capping protein (actin filament), gelsolin-like	Hs.516155
CARD6	caspase recruitment domain family, member 6	Hs.200242
CAT	catalase	Hs.502302
CD9	CD9 molecule	Hs.114286
CLU	clusterin	Hs.436657
CRABP2	cellular retinoic acid binding protein 2	Hs.405662
CTH	cystathionase (cystathionine gamma-lyase)	Hs.19904
CYP1B1	cytochrome P450, family 1, subfamily B, polypeptide 1	Hs.154654
DDIT4	DNA-damage-inducible transcript 4	Hs.523012
DHRS3	dehydrogenase/reductase (SDR family) member 3	Hs.289347
DOCK11	dedicator of cytokinesis 11	Hs.368203
ECH1	enoyl Coenzyme A hydratase 1, peroxisomal	Hs.196176
ELF3	E74-like factor 3 (ets domain transcription factor, epithelial-specific)	Hs.67928
EPHX1	epoxide hydrolase 1, microsomal (xenobiotic)	Hs.89649
FA2H	fatty acid 2-hydroxylase	Hs.461329
FADS2	fatty acid desaturase 2	Hs.502745
FAM26B	family with sequence similarity 26, member B	Hs.241545
FAM46A	family with sequence similarity 46, member A	Hs.10784
FAM46A	family with sequence similarity 46, member A	Hs.10784
FLJ13236	hypothetical protein FLJ13236	Hs.170298
FLJ20273	RNA-binding protein	Hs.518727
FLJ20920	hypothetical protein FLJ20920	Hs.288959
FLJ22675	hypothetical gene supported by AK026328	Hs.232604
GLRX	glutaredoxin (thioltransferase)	Hs.28988
GNG11	guanine nucleotide binding protein (G protein), gamma 11	Hs.83381
GNG2	guanine nucleotide binding protein (G protein), gamma 2	Hs.187772
GOT1	glutamic-oxaloacetic transaminase 1, soluble (aspartate aminotransferase 1)	Hs.500756
GPNMB	glycoprotein (transmembrane) nmb	Hs.190495
GSN	gelsolin (amyloidosis, Finnish type)	Hs.522373
HDLBP	high density lipoprotein binding protein (vigilin)	Hs.471851
HLA-G	HLA-G histocompatibility antigen, class I, G	Hs.512152
IDH1	isocitrate dehydrogenase 1 (NADP+), soluble	Hs.11223
IFI35	interferon-induced protein 35	Hs.50842
IFIT1	interferon-induced protein with tetratricopeptide repeats 1	Hs.20315
IGFBP6	insulin-like growth factor binding protein 6	Hs.274313
IL17RC	interleukin 17 receptor C	Hs.129959
INSIG1	insulin induced gene 1	Hs.520819
KIAA0274	KIAA0274	Hs.529959
KLF9	Kruppel-like factor 9	Hs.150557
KLK6	kallikrein 6 (neurosin, zyme)	Hs.79361
LAMA3	laminin, alpha 3	Hs.436367
LOC285943	hypothetical protein LOC285943	Hs.520721
LPAAT-THETA	lysophosphatidic acid acyltransferase theta	Hs.99196
LXN	latexin	Hs.478067
MAGED2	melanoma antigen family D, 2	Hs.4943
MAP1A	microtubule-associated protein 1A	Hs.194301
METTL7A	methyltransferase like 7A	Hs.288771
MGC16075	hypothetical protein MGC16075	Hs.334508
MMP2	matrix metallopeptidase 2 (gelatinase A, 72kDa gelatinase, 72kDa type IV collagenase)	Hs.513617
MVP	major vault protein	Hs.513488
NCOA7	nuclear receptor coactivator 7	Hs.171426

NDRG1	N-myc downstream regulated gene 1	Hs.372914
NEBL	nebulette	Hs.5025
NFE2	nuclear factor (erythroid-derived 2), 45kDa	Hs.75643
NFKBIA	nuclear factor of kappa light polypeptide gene enhancer in B-cells inhibitor, alpha	Hs.81328
NMNAT2	nicotinamide nucleotide adenylyltransferase 2	Hs.497123
NOV	nephroblastoma overexpressed gene	Hs.235935
NR1H3	nuclear receptor subfamily 1, group H, member 3	Hs.438863
NULL	NULL	Hs.535320
OAS1	2',5'-oligoadenylate synthetase 1, 40/46kDa	Hs.524760
OLIG2	oligodendrocyte lineage transcription factor 2	Hs.176977
OPTN	optineurin	Hs.332706
P4HA2	procollagen-proline, 2-oxoglutarate 4-dioxygenase (proline 4-hydroxylase), alpha polypeptide II	Hs.519568
PC	pyruvate carboxylase	Hs.89890
PDCD4	programmed cell death 4 (neoplastic transformation inhibitor)	Hs.232543
PGM1	phosphoglucomutase 1	Hs.1869
PIR	pirin (iron-binding nuclear protein)	Hs.495728
POR	P450 (cytochrome) oxidoreductase	Hs.354056
PPARA	peroxisome proliferative activated receptor, alpha	Hs.103110
PPARA	peroxisome proliferative activated receptor, alpha	Hs.275711
PPARG	peroxisome proliferative activated receptor, gamma	Hs.162646
PPP1R1A	protein phosphatase 1, regulatory (inhibitor) subunit 1A	Hs.505662
PSMB10	proteasome (prosome, macropain) subunit, beta type, 10	Hs.9661
PTGES	prostaglandin E synthase	Hs.146688
QPRT	quinolinate phosphoribosyltransferase (nicotinate-nucleotide pyrophosphorylase (carboxylating))	Hs.513484
RAB27B	RAB27B, member RAS oncogene family	Hs.25318
RAB31	RAB31, member RAS oncogene family	Hs.99528
RAB7B	RAB7B, member RAS oncogene family	Hs.534612
RAP1GAP	RAP1 GTPase activating protein	Hs.148178
RARRES3	retinoic acid receptor responder (tazarotene induced) 3	Hs.17466
RASSF4	Ras association (RalGDS/AF-6) domain family 4	Hs.522895
S100A3	S100 calcium binding protein A3	Hs.433168
S100A4	S100 calcium binding protein A4	Hs.81256
S100A6	S100 calcium binding protein A6	Hs.275243
SCD	stearoyl-CoA desaturase (delta-9-desaturase)	Hs.368641
SCNN1A	sodium channel, nonvoltage-gated 1 alpha	Hs.130989
SDCBP	syndecan binding protein (syntenin)	Hs.200804
SERPINB1	serpin peptidase inhibitor, clade B (ovalbumin), member 1	Hs.381167
SLC2A3	solute carrier family 2 (facilitated glucose transporter), member 3	Hs.419240
SLC9A3R1	solute carrier family 9 (sodium/hydrogen exchanger), member 3 regulator 1	Hs.396783
SMAD3	SMAD, mothers against DPP homolog 3 (Drosophila)	Hs.99843
SNCG	synuclein, gamma (breast cancer-specific protein 1)	Hs.349470
SSBP2	single-stranded DNA binding protein 2	Hs.102735
STC1	stanniocalcin 1	Hs.25590
SULF2	sulfatase 2	Hs.162016
SYNPO	synaptopodin	Hs.435228
TCN2	transcobalamin II; macrocytic anemia	Hs.417948
TGFBR2	transforming growth factor, beta receptor II (70/80kDa)	Hs.82028
TIMP3	TIMP metallopeptidase inhibitor 3 (Sorsby fundus dystrophy, pseudoinflammatory)	Hs.297324
TIMP4	TIMP metallopeptidase inhibitor 4	Hs.549073
TLR3	toll-like receptor 3	Hs.29499
TLR4	toll-like receptor 4	Hs.174312
TMEM140	transmembrane protein 140	Hs.521215
TNFRSF9	tumor necrosis factor receptor superfamily, member 9	Hs.193418
TNIK	TRAF2 and NCK interacting kinase	Hs.34024
TP53INP2	tumor protein p53 inducible nuclear protein 2	Hs.516994
TPBG	trophoblast glycoprotein	Hs.82128
TPD52L1	tumor protein D52-like 1	Hs.201482
TSC22D1	TSC22 domain family, member 1	Hs.507916
TSC22D3	TSC22 domain family, member 3	Hs.522074
UBE2L6	ubiquitin-conjugating enzyme E2L 6	Hs.425777
VAMP5	vesicle-associated membrane protein 5 (myobrevin)	Hs.172684
VAV3	vav 3 oncogene	Hs.267659
WNT6	wingless-type MMTV integration site family, member 6	Hs.29764

Genes upregulated in all three mesothelioma cell lines (M28, REN, VAMT) in spheroids compared to monolayers with Bonferoni adjusted $P<0.05$.
