# Supplementary material for: Analysis of Gene Expression in 3D Spheroids Highlights a Survival Role for ASS1 in Mesothelioma
Source: PLoS One. 2016 Mar 16;11(3):e0150044. doi: 10.1371/journal.pone.0150044 (PMC4794185; doi:10.1371/journal.pone.0150044)
Supplement: S2 Table — The table shows the 71 genes downregulated in 3D spheroids grown from M28, REN and VAMT cell lines. (PDF) [file pone.0150044.s004.pdf]

Table S2 | **Downregulated genes in mesothelioma spheroids**

Gene Symbol	Description	Unigene ID
AADACL1	arylacetamide deacetylase-like 1	Hs.444099
ADRB2	adrenergic, beta-2-, receptor, surface	Hs.2551
AMOTL2	angiomotin like 2	Hs.426312
ANKRD1	ankyrin repeat domain 1 (cardiac muscle)	Hs.448589
ANKRD13A	ankyrin repeat domain 13A	Hs.528703
ANKRD2	ankyrin repeat domain 2 (stretch responsive muscle)	Hs.73708
ANXA3	annexin A3	Hs.480042
AXL	AXL receptor tyrosine kinase	Hs.466791
C12orf24	chromosome 12 open reading frame 24	Hs.436618
C1orf33	chromosome 1 open reading frame 33	Hs.463797
C1orf79	chromosome 1 open reading frame 79	Hs.40092
CAD	carbamoyl-phosphate synthetase 2, aspartate transcarbamylase, and dihydroorotase	Hs.377010
CCBE1	collagen and calcium binding EGF domains 1	Hs.34333
CCDC85A	coiled-coil domain containing 85A	Hs.117136
CGI-115	CGI-115 protein	Hs.408101
CTGF	connective tissue growth factor	Hs.410037
CYR61	cysteine-rich, angiogenic inducer, 61	Hs.8867
DIO2	deiodinase, iodothyronine, type II	Hs.202354
DLX2	distal-less homeobox 2	Hs.419
EDN1	endothelin 1	Hs.511899
EPHB2	EPH receptor B2	Hs.523329
F3	coagulation factor III (thromboplastin, tissue factor)	Hs.62192
FGF2	fibroblast growth factor 2 (basic)	Hs.284244
FGFR2	fibroblast growth factor receptor 2	Hs.533683
FJX1	four jointed box 1 (Drosophila)	Hs.39384
FST	follistatin	Hs.9914
GADD45A	growth arrest and DNA-damage-inducible, alpha	Hs.80409
GTPBP4	GTP binding protein 4	Hs.215766
IL11	interleukin 11	Hs.467304
KIAA0690	KIAA0690	Hs.434251
KISS1	KiSS-1 metastasis-suppressor	Hs.95008
KRT33B	keratin 33B	Hs.32950
LHB	luteinizing hormone beta polypeptide	Hs.154704
LOC56902	putatative 28 kDa protein	Hs.262858
MKI67IP	MKI67 (FHA domain) interacting nucleolar phosphoprotein	Hs.367842
MYBL1	v-myb myeloblastosis viral oncogene homolog (avian)-like 1	Hs.445898
MYC	v-myc myelocytomatosis viral oncogene homolog (avian)	Hs.202453
NEXN	nexilin (F actin binding protein)	Hs.22370
NIP7	nuclear import 7 homolog (S. cerevisiae)	Hs.501513
NPPB	natriuretic peptide precursor B	Hs.219140
NT5DC3	5'-nucleotidase domain containing 3	Hs.48428
NT5E	5'-nucleotidase, ecto (CD73)	Hs.153952
NULL	NULL	Hs.82254
ODC1	ornithine decarboxylase 1	Hs.467701
OXTR	oxytocin receptor	Hs.2820
POLR3G	polymerase (RNA) III (DNA directed) polypeptide G (32kD)	Hs.282387
POLR3G	polymerase (RNA) III (DNA directed) polypeptide G (32kD)	Hs.282387
PPAT	phosphoribosyl pyrophosphate amidotransferase	Hs.331420
PPRC1	peroxisome proliferative activated receptor, gamma, coactivator-related 1	Hs.533551
PWP2H	PWP2 periodic tryptophan protein homolog (yeast)	Hs.449076
RAD18	RAD18 homolog (S. cerevisiae)	Hs.375684
RBM24	RNA binding motif protein 24	Hs.519904
RGS7	regulator of G-protein signalling 7	Hs.130171
RRS1	RRS1 ribosome biogenesis regulator homolog (S. cerevisiae)	Hs.71827
SCHIP1	schwannomin interacting protein 1	Hs.134665
SEC23B	Sec23 homolog B (S. cerevisiae)	Hs.369373
SERPINE1	serpin peptidase inhibitor, clade E (nexin, plasminogen activator inhibitor type 1), member 1	Hs.414795
SNORD22	small nucleolar RNA, C/D box 22	Hs.433345
STYK1	serine/threonine/tyrosine kinase 1	Hs.24979
TMEM166	transmembrane protein 166	Hs.549210
TMEM16B	transmembrane protein 16B	Hs.148970
UAP1	UDP-N-acteylglucosamine pyrophosphorylase 1	Hs.492859
WNT5B	wingless-type MMTV integration site family, member 5B	Hs.306051
WWC1	WW, C2 and coiled-coil domain containing 1	Hs.484047
YRDC	yrdC domain containing (E. coli)	Hs.301564
ZC3H8	zinc finger CCCH-type containing 8	Hs.418416
ZFP30	zinc finger protein 30 homolog (mouse)	Hs.116622
ZFP36L2	zinc finger protein 36, C3H type-like 2	Hs.78909
ZNF569	zinc finger protein 569	Hs.511848
ZNF695	zinc finger protein 695	Hs.161840
ZNF714	zinc finger protein 714	Hs.466291

Genes downregulated in all three mesothelioma cell lines (M28, REN, VAMT) in spheroids compared to monolayers with Bonferoni adjusted $P<0.05$.
