# Supplementary material for: Analysis of Gene Expression in 3D Spheroids Highlights a Survival Role for ASS1 in Mesothelioma
Source: PLoS One. 2016 Mar 16;11(3):e0150044. doi: 10.1371/journal.pone.0150044 (PMC4794185; doi:10.1371/journal.pone.0150044)
Supplement: S3 Table — The table shows the top functions altered in 3D in the signaling families: molecular and cellular functions, physiological system development and function, and diseases and disorders. The pathways affected in the different categories are shown in the table, together with p value and number of differentially expressed genes in each pathway (Count). A comprehensive analysis of the functions affected by the differentially-expressed genes did not reveal a single suppressed or activated pathway. (PDF) [file pone.0150044.s005.pdf]

Table S3 | **Ingenuity top diseases and bio functions**

**Molecular and Cellular Functions**		
**Category**	**PValue**	**Count**
Cellular Growth and Proliferation	5,81E-18 - 3,51E-04	108
Cellular Movement	1,28E-17 - 4,42E-04	74
Cell Death and Survival	1,87E-15 - 4,18E-04	95
Cellular Development	1,17E-13 - 3,51E-04	88
Cell Morphology	1,28E-08 - 4,42E-04	65
**Physiological System Development and Function**		
**Category**	**PValue**	**Count**
Tumor Morphology	3,54E-13 - 3,59E-04	47
Immune Cell Trafficking	6,03E-11 - 4,21E-04	39
Tissue Morphology	2,53E-10 - 3,61E-04	65
Cardiovascular System Development and Function	8,16E-10 - 3,77E-04	51
Organismal Development	8,16E-10 - 3,45E-04	69
**Diseases and Disorders**		
**Category**	**PValue**	**Count**
Cancer	2,05E-18 - 3,82E-04	151
Dermatological Diseases and Conditions	2,71E-13 - 3,39E-04	48
Developmental Disorder	4,51E-13 - 3,61E-04	31
Organismal Injury and Abnormalities	3,68E-12 - 3,61E-04	96
Reproductive System Disease	3,68E-12 - 3,59E-04	73

Ingenuity Top Molecular and Cellular Funtions, Physiological System Development and Function and Diseases and Disorders for DE genes in mesothelioma spheroids.
