# Supplementary material for: Analysis of Gene Expression in 3D Spheroids Highlights a Survival Role for ASS1 in Mesothelioma
Source: PLoS One. 2016 Mar 16;11(3):e0150044. doi: 10.1371/journal.pone.0150044 (PMC4794185; doi:10.1371/journal.pone.0150044)
Supplement: S4 Table — The table shows the DAVID functional annotation chart with p<0.01 for the differentially expressed genes in 3D. (PDF) [file pone.0150044.s006.pdf]

Table S4 | **DAVID functional annotation chart**

Category	Term	Count	%	PValue	Fold Enrichment
GOTERM_BP_FAT	GO:0042127~regulation of cell proliferation	24	11.8	6.29E-05	2.53
GOTERM_BP_FAT	GO:0042060~wound healing	11	5.4	1.00E-04	4.78
GOTERM_BP_FAT	GO:0032101~regulation of response to external stimulus	10	4.9	1.25E-04	5.22
INTERPRO	IPR017891:Insulin-like growth factor binding protein, N-terminal	4	2.0	2.71E-04	30.02
GOTERM_BP_FAT	GO:0045596~negative regulation of cell differentiation	11	5.4	2.74E-04	4.23
GOTERM_BP_FAT	GO:0010888~negative regulation of lipid storage	4	2.0	3.43E-04	27.66
GOTERM_BP_FAT	GO:0010745~negative regulation of foam cell differentiation	4	2.0	3.43E-04	27.66
SP_PIR_KEYWORDS	endoplasmic reticulum	19	9.3	4.84E-04	2.55
GOTERM_BP_FAT	GO:0009611~response to wounding	17	8.3	6.25E-04	2.66
INTERPRO	IPR006208:Cystine knot	4	2.0	6.68E-04	22.51
GOTERM_BP_FAT	GO:0010033~response to organic substance	20	9.8	1.01E-03	2.30
GOTERM_CC_FAT	GO:0044421~extracellular region part	23	11.3	1.07E-03	2.11
SMART	SM00121:IB	4	2.0	1.11E-03	18.91
UP_SEQ_FEATURE	domain:IGFBP N-terminal	4	2.0	1.13E-03	19.02
UP_SEQ_FEATURE	region of interest:Heparin-binding	4	2.0	1.13E-03	19.02
INTERPRO	IPR012395:IGFBP-related, CNN	3	1.5	1.19E-03	54.03
UP_SEQ_FEATURE	binding site:Substrate	11	5.4	1.19E-03	3.52
GOTERM_BP_FAT	GO:0051493~regulation of cytoskeleton organization	8	3.9	1.24E-03	4.88
INTERPRO	IPR000867:Insulin-like growth factor-binding protein, IGFBP	4	2.0	1.32E-03	18.01
GOTERM_BP_FAT	GO:0031099~regeneration	6	2.9	1.40E-03	7.22
GOTERM_BP_FAT	GO:0050818~regulation of coagulation	5	2.5	1.42E-03	10.12
GOTERM_BP_FAT	GO:0042445~hormone metabolic process	7	3.4	1.71E-03	5.48
GOTERM_BP_FAT	GO:0046165~alcohol biosynthetic process	5	2.5	1.85E-03	9.43
GOTERM_BP_FAT	GO:0010883~regulation of lipid storage	4	2.0	1.91E-03	15.81
PIR_SUPERFAMILY	PIRSF036495:IGFBP_rP_CNN	3	1.5	2.05E-03	40.71
GOTERM_BP_FAT	GO:0010887~negative regulation of cholesterol storage	3	1.5	2.07E-03	41.50
GOTERM_CC_FAT	GO:0005730~nucleolus	18	8.8	2.19E-03	2.27
GOTERM_BP_FAT	GO:0010817~regulation of hormone levels	8	3.9	2.27E-03	4.40
GOTERM_BP_FAT	GO:0001558~regulation of cell growth	9	4.4	2.33E-03	3.85
GOTERM_BP_FAT	GO:0008219~cell death	19	9.3	2.42E-03	2.19
GOTERM_BP_FAT	GO:0019748~secondary metabolic process	6	2.9	2.56E-03	6.30
GOTERM_BP_FAT	GO:0016265~death	19	9.3	2.61E-03	2.18
GOTERM_BP_FAT	GO:0051240~positive regulation of multicellular organismal process	10	4.9	2.70E-03	3.40
SP_PIR_KEYWORDS	duplication	9	4.4	2.72E-03	3.78
GOTERM_MF_FAT	GO:0005520~insulin-like growth factor binding	4	2.0	2.89E-03	13.76
SP_PIR_KEYWORDS	metalloprotease inhibitor	3	1.5	2.89E-03	35.89
GOTERM_BP_FAT	GO:0008202~steroid metabolic process	9	4.4	2.99E-03	3.70
SP_PIR_KEYWORDS	lipoprotein	16	7.8	3.00E-03	2.38
PIR_SUPERFAMILY	PIRSF036495:IGFBP-related protein, CNN type	3	1.5	3.05E-03	33.93
GOTERM_BP_FAT	GO:0010743~regulation of foam cell differentiation	4	2.0	3.19E-03	13.28
SP_PIR_KEYWORDS	polymorphism	140	68.6	3.38E-03	1.16
SP_PIR_KEYWORDS	metalloenzyme inhibitor	3	1.5	3.69E-03	31.90
GOTERM_BP_FAT	GO:0008285~negative regulation of cell proliferation	12	5.9	4.15E-03	2.76
GOTERM_BP_FAT	GO:0032956~regulation of actin cytoskeleton organization	6	2.9	4.28E-03	5.60
GOTERM_CC_FAT	GO:0005615~extracellular space	17	8.3	4.44E-03	2.19
SP_PIR_KEYWORDS	calcium binding	6	2.9	4.44E-03	5.57
GOTERM_BP_FAT	GO:0033043~regulation of organelle organization	9	4.4	4.62E-03	3.44
GOTERM_BP_FAT	GO:0052200~response to host defenses	3	1.5	4.86E-03	27.66
GOTERM_BP_FAT	GO:0052173~response to defenses of other organism during symbiotic interaction	3	1.5	4.86E-03	27.66
GOTERM_BP_FAT	GO:0075136~response to host	3	1.5	4.86E-03	27.66
GOTERM_BP_FAT	GO:0032970~regulation of actin filament-based process	6	2.9	4.93E-03	5.41
SP_PIR_KEYWORDS	microsome	6	2.9	5.02E-03	5.42
GOTERM_BP_FAT	GO:0032535~regulation of cellular component size	10	4.9	5.37E-03	3.06
GOTERM_CC_FAT	GO:0005792~microsome	9	4.4	5.39E-03	3.35
GOTERM_BP_FAT	GO:0010551~regulation of specific transcription from RNA polymerase II promoter	6	2.9	5.40E-03	5.30
GOTERM_BP_FAT	GO:0034754~cellular hormone metabolic process	5	2.5	5.41E-03	7.03
UP_SEQ_FEATURE	sequence variant	144	70.6	5.52E-03	1.14
GOTERM_MF_FAT	GO:0048551~metalloenzyme inhibitor activity	3	1.5	5.61E-03	25.79
GOTERM_MF_FAT	GO:0010576~metalloenzyme regulator activity	3	1.5	5.61E-03	25.79
GOTERM_MF_FAT	GO:0008191~metalloendopeptidase inhibitor activity	3	1.5	5.61E-03	25.79
GOTERM_BP_FAT	GO:0051051~negative regulation of transport	7	3.4	5.68E-03	4.30
SP_PIR_KEYWORDS	nadp	7	3.4	5.88E-03	4.29
UP_SEQ_FEATURE	domain:Leucine-zipper	6	2.9	6.02E-03	5.19
INTERPRO	IPR018170:Aldo/keto reductase, conserved site	3	1.5	6.25E-03	24.56
GOTERM_BP_FAT	GO:0045087~innate immune response	7	3.4	6.31E-03	4.21
GOTERM_BP_FAT	GO:0051094~positive regulation of developmental process	10	4.9	6.31E-03	2.99
GOTERM_CC_FAT	GO:0042598~vesicular fraction	9	4.4	6.39E-03	3.25
GOTERM_BP_FAT	GO:0042246~tissue regeneration	4	2.0	6.48E-03	10.37

GOTERM_MF_FAT	GO:0031406~carboxylic acid binding	7	3.4	6.51E-03	4.18
UP_SEQ_FEATURE	site:Lowers pKa of active site Tyr	3	1.5	6.71E-03	23.77
UP_SEQ_FEATURE	nucleotide phosphate-binding region:NADP	5	2.5	6.88E-03	6.60
GOTERM_BP_FAT	GO:0050878~regulation of body fluid levels	7	3.4	6.99E-03	4.12
GOTERM_BP_FAT	GO:0008217~regulation of blood pressure	6	2.9	6.99E-03	4.98
GOTERM_BP_FAT	GO:0044087~regulation of cellular component biogenesis	7	3.4	7.23E-03	4.09
GOTERM_BP_FAT	GO:0010885~regulation of cholesterol storage	3	1.5	7.30E-03	22.63
KEGG_PATHWAY	hsa00250:Alanine, aspartate and glutamate metabolism	4	2.0	7.55E-03	9.65
GOTERM_BP_FAT	GO:0048584~positive regulation of response to stimulus	9	4.4	7.56E-03	3.17
GOTERM_BP_FAT	GO:0002684~positive regulation of immune system process	9	4.4	7.93E-03	3.14
GOTERM_BP_FAT	GO:0006869~lipid transport	7	3.4	7.98E-03	4.01
GOTERM_BP_FAT	GO:0050778~positive regulation of immune response	7	3.4	7.98E-03	4.01
GOTERM_BP_FAT	GO:0040008~regulation of growth	11	5.4	8.00E-03	2.68
GOTERM_BP_FAT	GO:0048878~chemical homeostasis	14	6.9	8.61E-03	2.27
SP_PIR_KEYWORDS	innate immunity	5	2.5	8.89E-03	6.13
GOTERM_BP_FAT	GO:0030193~regulation of blood coagulation	4	2.0	9.01E-03	9.22
GOTERM_BP_FAT	GO:0034637~cellular carbohydrate biosynthetic process	5	2.5	9.37E-03	6.01
GOTERM_MF_FAT	GO:0005506~iron ion binding	10	4.9	9.52E-03	2.79
GOTERM_BP_FAT	GO:0046364~monosaccharide biosynthetic process	4	2.0	9.72E-03	8.97
GOTERM_BP_FAT	GO:0006631~fatty acid metabolic process	8	3.9	9.83E-03	3.35
GOTERM_BP_FAT	GO:0043408~regulation of MAPKKK cascade	6	2.9	9.97E-03	4.57
KEGG_PATHWAY	hsa05200:Pathways in cancer	11	5.4	9.99E-03	2.51

DAVID functional annotation chart with P<0.01 for DE genes in mesothelioma spheroids.
