# Supplementary material for: Analysis of Gene Expression in 3D Spheroids Highlights a Survival Role for ASS1 in Mesothelioma
Source: PLoS One. 2016 Mar 16;11(3):e0150044. doi: 10.1371/journal.pone.0150044 (PMC4794185; doi:10.1371/journal.pone.0150044)
Supplement: S5 Table — The table shows the ASS1 staining intensity of 88 mesotheliomas, categorized by their histotypes: epithelioid (46) and non epithelioid (42—sarcomatous and biphasic). The difference between the two groups was not statistically significant (p = 0.5662). (PDF) [file pone.0150044.s007.pdf]

Non-epithelioid	Epithelioid
0.000	0.167
0.333	0.333
0.333	0.500
0.333	0.500
0.500	0.500
0.667	0.667
0.833	0.833
1.000	0.833
1.000	0.833
1.000	0.833
1.167	1.000
1.167	1.000
1.167	1.000
1.167	1.000
1.167	1.167
1.333	1.167
1.333	1.167
1.333	1.333
1.500	1.333
1.500	1.333
1.500	1.333
1.500	1.333
1.667	1.333
1.667	1.333
1.667	1.500
1.667	1.500
1.667	1.500
1.667	1.500
1.833	1.500
1.833	1.667
1.833	1.667
1.833	1.667
1.833	1.750
2.000	1.750
2.000	1.750
2.000	1.833
2.000	1.833
2.000	1.833
2.000	1.833
2.333	2.000
2.333	2.000
2.500	2.500
	2.500
	2.500
	2.667
	2.833

Mann Whitney test
Non-epithelioid vs epithelioid
P value 0.5662
